# Structural determination of an antibody that specifically recognizes polyethylene glycol with a terminal methoxy group

**DOI:** 10.1038/s42004-022-00709-0

**Published:** 2022-08-01

**Authors:** Minh-Tram T. Nguyen, Yu-Chien Shih, Meng-Hsuan Lin, Steve R. Roffler, Chiao-Yu Hsiao, Tian-Lu Cheng, Wen-Wei Lin, En-Chi Lin, Yuh-Jyh Jong, Chin-Yuan Chang, Yu-Cheng Su

**Affiliations:** 1 Department of Biological Science and Technology, Center for Intelligent Drug Systems and Smart Bio-devices (IDS²B), National Yang Ming Chiao Tung University, Hsinchu, Taiwan; 2 Institute of Biomedical Sciences, Academia Sinica, Taipei, Taiwan; 3 Graduate Institute of Medicine, College of Medicine, Kaohsiung Medical University, Kaohsiung, Taiwan; 4 Department of Biomedical Science and Environmental Biology, Drug Development and Value Creation Research Center, Kaohsiung Medical University, Kaohsiung, Taiwan; 5 School of Post-Baccalaureate Medicine, College of Medicine, Kaohsiung Medical University, Kaohsiung, Taiwan; 6 Graduate Institute of Clinical Medicine, Kaohsiung Medical University, Kaohsiung, Taiwan; 7 Departments of Pediatrics and Laboratory Medicine, and Translational Research Center of Neuromuscular Diseases, Kaohsiung Medical University Hospital, Kaohsiung, Taiwan

**Keywords:** X-ray crystallography, Proteins

## Abstract

Covalent attachment of methoxy poly(ethylene) glycol (mPEG) to therapeutic molecules is widely employed to improve their systemic circulation time and therapeutic efficacy. mPEG, however, can induce anti-PEG antibodies that negatively impact drug therapeutic effects. However, the underlying mechanism for specific binding of antibodies to mPEG remains unclear. Here, we determined the first co-crystal structure of the humanized 15-2b anti-mPEG antibody in complex with mPEG, which possesses a deep pocket in the antigen-binding site to accommodate the mPEG polymer. Structural and mutational analyses revealed that mPEG binds to h15-2b via Van der Waals and hydrogen bond interactions, whereas the methoxy group of mPEG is stabilized in a hydrophobic environment between the V_H_:V_L_ interface. Replacement of the heavy chain hydrophobic V37 residue with a neutral polar serine or threonine residue offers additional hydrogen bond interactions with methoxyl and hydroxyl groups, resulting in cross-reactivity to mPEG and OH-PEG. Our findings provide insights into understanding mPEG-binding specificity and antigenicity of anti-mPEG antibodies.

## Abstract (web summary)

Methoxy polyethylene glycol (mPEG) is widely attached to drug molecules to improve their therapeutic efficacy, however mPEG can induce anti-PEG antibodies that negatively impact their therapeutic effects. Here, the authors determine the co-crystal structure of the humanized 15-2b anti-mPEG antibody with mPEG, providing insights into understanding mPEG-binding specificity and antigenicity of anti-mPEG antibodies.

## Introduction

PEGylation is a widely applied method that covalently conjugates therapeutics with methoxy polyethylene glycol (mPEG) for improving their pharmaceutical and pharmacokinetic properties^1–3^. mPEGylated therapeutics have been approved by the U.S. Food and Drug Administration, including small molecular drugs^4,5^, proteins^6–9^ and nanoparticles^10,11^. For example, low molecular weight mPEG can be conjugated with hydrophobic small molecular drugs^4,5^ to improve their water solubility and decrease systemic toxicity^4,5^. On the other hand, high molecular weight mPEG is often attached to the surface of therapeutic proteins to prolong their serum half-life and protect against proteolytic degradation^7–9^. Incorporation of mPEG molecules on nanoparticles such as Doxil (mPEG-liposomal doxorubicin) and COVID-19 mRNA vaccines (mPEG-containing lipid nanoparticles-mRNA) can reduce unwanted uptake by the reticuloendothelial system in vivo^10^ and prevent lipid nanoparticles-mRNA aggregation during storage as an aqueous dispersion^12^, respectively.

mPEG is a biocompatible, well-tolerated polymer but many preclinical and clinical studies report that mPEGylated therapeutic molecules can trigger anti-PEG antibody production leading to reduced therapeutic efficacy^13–15^. For instance, anti-PEG antibodies can form immune complexes with mPEGylated therapeutics to induce complement activation resulting in accelerated blood clearance (ABC) via uptake into macrophages in the liver^15,16^. In addition, drug encapsulated mPEGylated liposomes can be destabilized by anti-PEG antibody-mediated complement activation, resulting in rapid drug leakage and diminished anti-tumor activity^14,17^. Surprisingly, pre-existing anti-PEG antibodies have been discovered in healthy donors^18,19^, raising concerns for induction of severe allergic reactions in some individuals who receive mPEGylated therapeutics, including PEGylated protein drugs and COVID-19 mRNA vaccines^20,21^.

Anti-PEG antibodies can be classified into two broad groups depending on their binding specificity. Antibodies that bind to the repeating ethylene oxide repeats of PEG are termed “backbone-specific”, whereas antibodies that require the terminal methoxy group for binding are termed “methoxy-specific”^18,22,23^. Previous studies have shown that backbone-specific anti-PEG antibodies can negatively impact the biodistribution and therapeutic efficacy of mPEGylated medicines^13,14^. The crystal structures of two backbone-specific anti-PEG monoclonal antibodies (3.3 and 6.3) revealed that dimerization of these antibodies is essential for PEG-binding^24,25^. Although PEG used in most therapeutic medicines is terminated with a methoxy group, the clinical relevance of methoxy-specific anti-mPEG antibodies remains largely unknown. Previous studies demonstrated that foreign proteins modified with mPEG induce higher titers of anti-PEG antibodies as compared to proteins modified with PEG in animals^26,27^, but mPEG-modified liposomes trigger reduced accelerated blood clearance as compared to PEG-modified liposomes^28^. The mechanism of binding of methoxy-specific anti-mPEG antibodies is also unknown.

In this study, we elucidate the binding mode of the humanized h15-2b antibody, which selectively binds to mPEG. We describe the crystal structure of h15-2b Fab in complex with mPEG. Structure-guided mutagenesis was performed to define crucial amino acid residues involved in binding to mPEG. Our findings provide new understandings of anti-PEG antibody specificity and mPEG antigenicity.

## Results

### h15-2b Fab specifically binds to mPEG via the terminal methoxy group

**Fig. 1:**
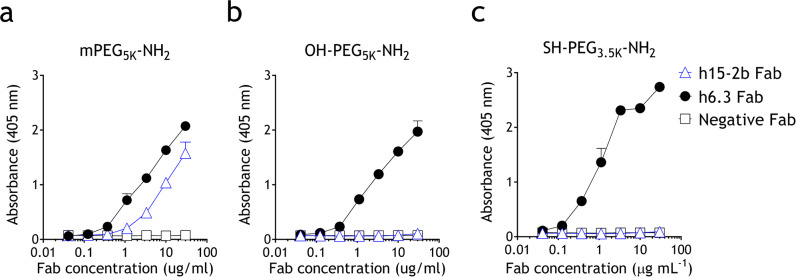
Characterization of anti-PEG Fab binding to immobilized PEG molecules. Microplate wells coated with amino-PEG molecules terminated (**a**) methoxy, (**b**) hydroxyl, or (**c**) thiol groups were incubated with graded concentrations of backbone-specific (h6.3, black circle) or methoxy-specific (h15-2b, blue open triangle) anti-PEG Fabs or anti-GFP Fab (negative control, open square). After washing, the Fab binding was determined by adding HRP-conjugated goat anti-human F(ab’)_2_ fragment specific antibodies, followed by ABTS substrate. The results show the mean absorbance values (405 nm) ± standard deviation (*n* = 3). The data are representative of three independent experiments.

Anti-PEG antibodies can be classified as backbone-specific and methoxy-specific. For instance, humanized 6.3 (h6.3) binds to the repeating ethylene oxide repeats in the PEG backbone, whereas h15-2b binds to PEG terminated with a methoxy moiety^18,22^. To examine the binding specificity of these anti-PEG antibodies, PEG molecules with various end groups, including mPEG_5K_-NH_2,_ OH-PEG_5K_-NH_2_, and SH-PEG_3.5K_-NH_2_, which contain a terminal methoxy, hydroxyl, or thiol groups, respectively, were immobilized on the surface of ELISA plates via the amine group^18^. Comparison of the binding specificity of recombinant Fab fragments derived from h6.3 or h15-2b as well as a negative control human anti-GFP Fab shows that h15-2b Fab strongly binds to mPEG_5K_-NH_2_ but not to OH-PEG_5K_-NH_2_ or SH-PEG_3.5K_-NH_2_ (Fig. 1), demonstrating that h15-2b displays selectivity for the terminal methoxy group of mPEG. By contrast, h6.3 Fab binds to all variant PEG molecules, indicating that h6.3 Fab binds to the repeating subunits of the PEG backbone (Fig. 1).

### h15-2b Fab complex with mPEG deep in the V_H_:V_L_ interface

**Fig. 2:**
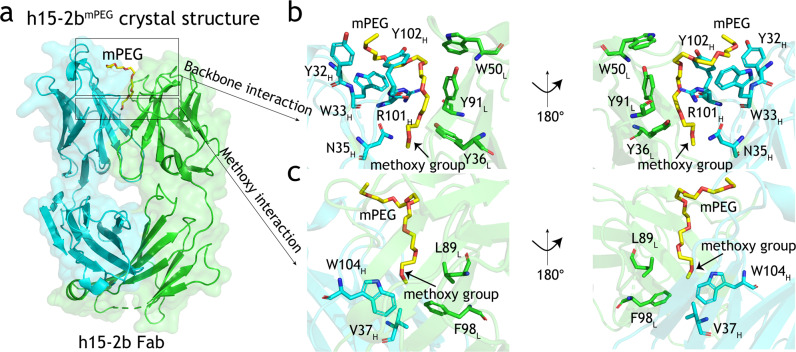
Structural analysis of critical residues for the interaction of h15-2b Fab and mPEG. **a** Overall crystal structure of the mPEG molecule and anti-mPEG h15-2b Fab complex (Light chain in green; Heavy chain in cyan). The mPEG fragment bound by h15-2b Fab is shown as a yellow stick model. Superposition of (**b**) mPEG backbone-specific binding residues and (**c**) methoxy-specific binding residues in the h15-2b^mPEG^ crystal structure are colored (Light chain in green; Heavy chain in cyan) and displayed in a transparent mode. The methoxy group of mPEG is indicated with an arrow.

**Fig. 3:**
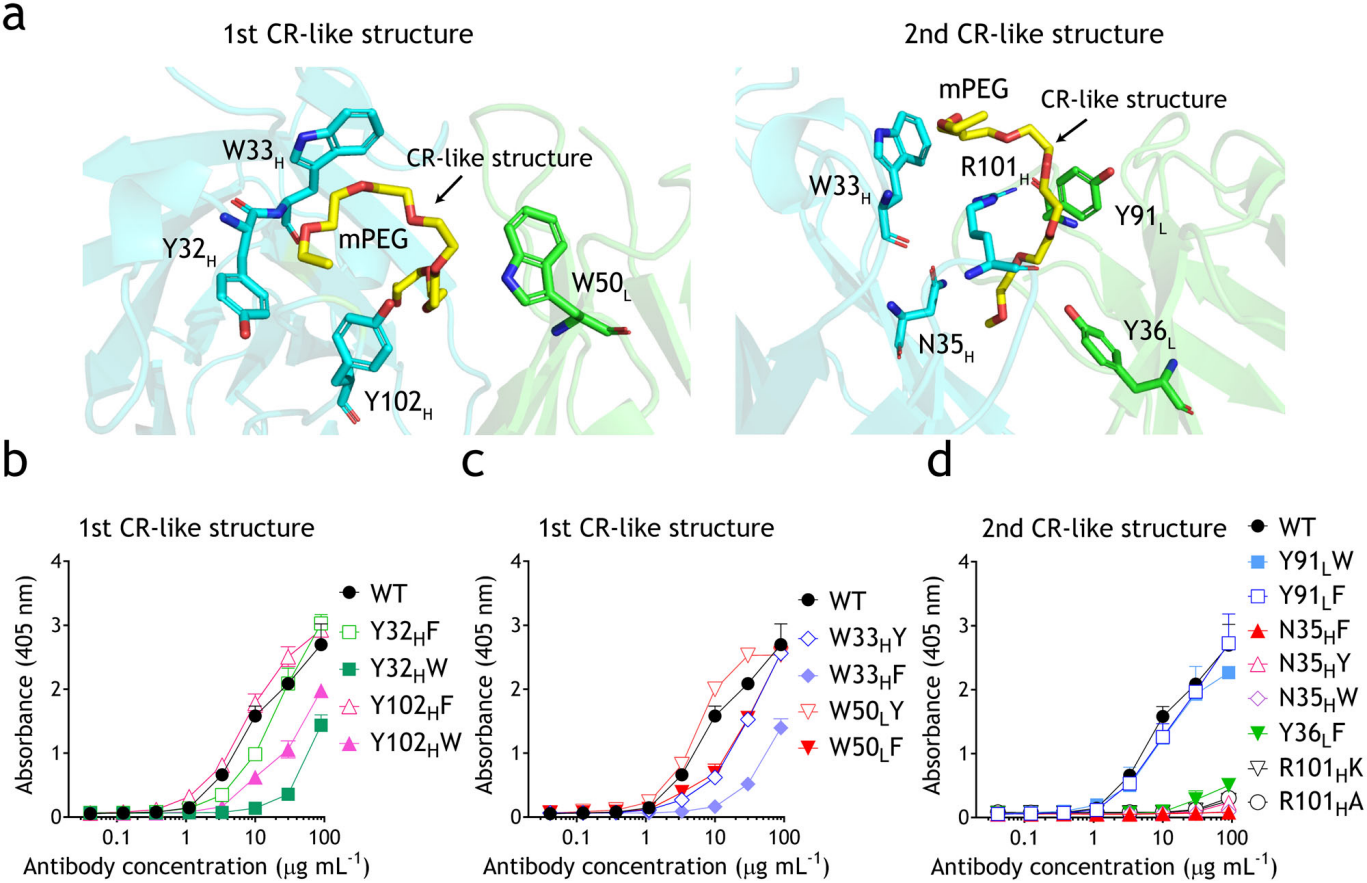
Comparison of h15-2b Fab variants for mPEG interaction. **a** CR-like structures of mPEG and anti-mPEG h15-2b Fab complex (Light chain in green; Heavy chain in cyan). The mPEG fragment bound by h15-2b Fab is shown as a yellow stick model. **b**–**d** Microplate wells coated with mPEG_5K_-NH_2_ were incubated with graded concentrations of parental h15-2b or relevant variants corresponding to CR-like structures of mPEG interaction. After 1 h, the wells were washed, and antibody binding was determined by adding HRP-conjugated goat anti-human F(ab’)_2_ fragment specific antibodies, followed by ABTS substrate. The results show the mean absorbance values (405 nm) ± standard deviation (*n* = 3). The data are representative of three independent experiments.

**Fig. 4:**
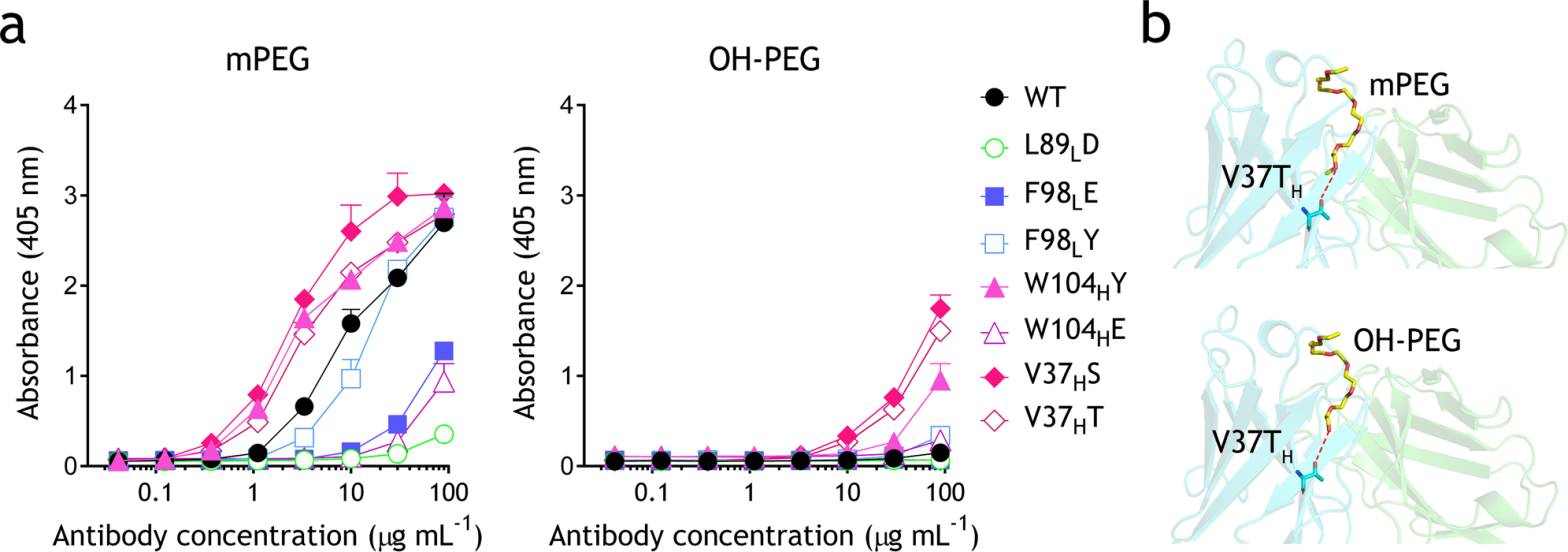
Specificity of h15-2b Fab variants against methoxy- and hydroxyl-PEG molecules. **a** Microplate wells coated with mPEG_5K_-NH_2_ and OH-PEG_5K_-NH_2_ were incubated with graded concentrations of parental h15-2b or relevant variants corresponding to methoxy group interacting residues. After 1 h, the wells were washed, and antibody binding was determined by adding HRP-conjugated goat anti-human F(ab’)_2_ fragment specific antibodies, followed by ABTS substrate. The results show the mean absorbance values (405 nm) ± standard deviation (*n* = 3). The data are representative of three independent experiments. **b** Simulated binding models of mPEG or OH-PEG (yellow sticks) interacting with the V37_H_T variant. Hydrogen bonds are indicated by red dashes.

We performed X-ray crystallography to determine the three-dimensional structure of h15-2b^mPEG^ complex. The h15-2b^mPEG^ complex was crystallized in an orthorhombic space group *P2*_*1*_ with the unit cell dimensions: *a* = 63.99 Å, *b* = 112.29 Å, *c* = 158.73 Å. The crystal structure of h15-2b^mPEG^ was determined by the molecular replacement (MR) method using the structure of the anti-ErbB2 Fab2C4 (Protein Data bank entry 1L7I) as the search model^29^. The final model of h15-2b^mPEG^ was refined to a resolution of 2.08 Å with an *R* factor of 21.1% and an *R*_free_ of 24.5%. Data processing and refinement statistics are given in Table 1. The structure of h15-2b^mPEG^ contains four copies of h15-2b Fab in an asymmetric unit, which all share nearly identical structures with small root-mean-square deviations (rmsds) of 0.161–0.417 Å for Cα atom superposition (Supplementary Fig. 1a; Supplementary Data 1). The light chains of the four copies of h15-2b Fab had varying electron density from the N- to C-termini, with the starting from the first residue and ending between residues 211 and 213. The heavy chains of the four copies of h15-2b Fab could be reliably traced between residues 1 and 214–216, with the region between residues 127 and 135 lacking significant density. Each of h15-2b Fabs shows that a hook-shaped mPEG fragment corresponding to 7 ethylene oxide subunits (about 300 Da) was bound into the pocket formed by the V_H_ and V_L_ domains of h15-2b Fab (Fig. 2a). The superposition of the four copies of h15-2b Fab reveals that the binding poses of the mPEG fragments are almost identical (Supplementary Fig. 1b). It forms a van der Waals network with aromatic side chains in Y32_H_, W33_H_, and Y102_H_ from the heavy chain, and Y36_L_, W50_L_, and Y91_L_ from the light chain (Fig. 2b). In addition, the PEG backbone wraps around R101_H_. The terminal methoxy group of the mPEG fragment is buried in the V_H_:V_L_ interface pocket, where V37_H_, W104_H_, L89_L_, and F98_L_ create a hydrophobic environment to accommodate the methoxy group (Fig. 2c). In this study, one of the h15-2b Fab structures (Chain A for light chain and Chain B for heavy chain in the atomic coordinates of h15-2b^mPEG^) was utilized for figure generation (Figs. 2–4).

**Table 1:** Data collection and refinement statistics.

	15-2b^mPEG^
**Data collection**	
Space group	P2_1_
Cell dimensions	
* a*, *b*, *c* (Å)	63.99, 112.29, 158.73
α, β, γ (°)	90.00, 90.00, 90.00
Resolution (Å)	30.00–2.08 (2.15–2.08)
*R*_sym_ or *R*_merge_	5.8 (49.2)
*I* / σ*I*	24.8 (2.7)
Completeness (%)	98.5 (93.1)
Redundancy	4.8 (4.4)
**Refinement**	
Resolution (Å)	30.00–2.08
No. reflections	131932
*R*_work_ / *R*_free_	0.211/0.245
No. atoms	
Protein	12890
Ligand/ion	80
Water	692
*B*-factors	
Protein	30.8
Ligand/ion	38.4
Water	31.1
R.m.s. deviations	
Bond lengths (Å)	0.0048
Bond angles (°)	1.358

It should be noted that the co-crystal structure of h15-2b Fab and mPEG was crystallized by using a 6:1 molar ratio of OH-PEG_6K_ (15 mmol L^−1^) and mPEG_2K_ (2.5 mmol L^−1^) as a cryoprotectant and an antigen, respectively. Although we demonstrated the specificity of h15-2b toward mPEG but not OH-PEG in Fig. 1, it raises a question of whether h15-2b Fab forms a complex with mPEG_2K_ or more abundant OH-PEG_6K_ during protein crystallization. To address this issue, we performed competitive ELISA with OH-PEG_6K_ and mPEG_2K_. Microplates coated with mPEG_2K_-NH_2_ or TNF-α (2 µmol L^−1^) were competed with either OH-PEG_6K_ or mPEG_5K_ (12 µmol L^−1^) in the presence of a fixed amount of Fab. A detailed experimental process can be found in Supplementary Methods. We found that both OH-PEG_6K_ and mPEG_5K_ blocked the binding of 6.3 Fab (backbone-specific) to immobilized mPEG_2K_-NH_2_ in a dose-dependent manner (Supplementary Fig. 2a, b). By contrast, OH-PEG_6K_ is a poor competitor, while mPEG_5K_ is a strong competitor for binding of the h15-2b Fab (methoxy-specific) to immobilized mPEG_2K_-NH_2_. Therefore, the competitive ELISA results suggest that h15-2b Fab can specifically bind to mPEG and tolerate the presence of 6-fold molar excess of OH-PEG_6K_ (Supplementary Fig. 2). Furthermore, neither OH-PEG_6K_ nor mPEG_5K_ affects the binding of an anti-TNF-α control Fab (Humira) to immobilized TNF-α, demonstrating the specificity of the competition reaction (Supplementary Fig. 2c, d). Taken together, h15-2b is more likely to form a complex with mPEG_2K_ instead of OH-PEG_6K_.

### Characterization of critical residues for h15-2b mPEG interaction

To verify critical amino acid residues that interact with the mPEG chain, we performed site-directed mutagenesis^30^ focusing on the amino acids around the backbone of mPEG. Previous studies suggest that aromatic residues play major roles in binding crown ether (CR)-like structure in the PEG backbone^24,25^. Since H-bonds also provide a significant intermolecular force for interaction, we investigated the importance of H-bonding interactions between aromatic side chains and the ether oxygen atoms of mPEG. Thus, we mutated Y32_H_, W33_H_, N35_H_, Y102_H_, Y36_L_, W50_L,_ and Y91_L_ into different aromatic residues (Y or W or F) that possess aromatic rings with or without the capability to form H-bonds.

Aromatic residues, including Y32_H_, W33_H_, Y102_H_, and W50_L,_ clamp the first semicircular CR-like structure of mPEG (Fig. 3a). Mutation of Y32_H_ and Y102_H_ to phenylalanine (Y32_H_F and Y102_H_F) produce similar mPEG-binding activity as parental h15-2b, suggesting that the benzene ring side-chain is critical for mPEG interaction through hydrophobic forces but not through H-bonds (Fig. 3b; Supplementary Table 1). By contrast, Y32_H_W and Y102_H_W exhibited impaired mPEG-binding activity, indicating that the bulky indole group of tryptophan might negatively impact the mPEG interaction (Fig. 3b; Supplementary Table 1). Mutating W33_H_ and W50_L_ to tyrosine resulted in similar or minor decreased mPEG-binding activity, whereas W33_H_F and W50_L_F displayed a clear reduction of mPEG-binding, suggesting that the indole ring of W33_H_ and W50_L_ might participate in mPEG-binding via H-bonds (Fig. 3c; Supplementary Table 1). The second semicircular CR-like structure is clamped by W33_H,_ N35_H_, Y36_L,_ and Y91_L_ (Fig. 3a). Both Y91_L_F and Y91_L_W showed similar mPEG-binding activity as the parental h15-2b, indicating that the aromatic side chain plays a major role to contact the mPEG chain without the need of H-bonding (Fig. 3d; Supplementary Table 1). Structural analysis also showed that the phenolic hydroxyl group of Y36_L_ forms an H-bond with the ether oxygen atoms of mPEG. Indeed, mutation of Y36_L_ to phenylalanine (Y36_L_F) completely abolished mPEG-binding activity, indicating an important role of Y36_L_ for mPEG interaction (Fig. 3d; Supplementary Table 1). In addition, N35_H_ also forms an H-bond with the ether oxygen atoms of mPEG. Thus, mutation of N35_H_ to aromatic residues leads to the elimination of the mPEG-binding activity (Fig. 3d; Supplementary Table 1). These results suggest that the flexible mPEG chain is captured by h15-2b Fab through van der Waals forces from aromatic side chains that clamp the PEG backbone with possible hydrogen bond formation between the hydroxyl group or indole nitrogen atom of Y36_L_, W33 _H,_ and W50_L_ and oxygen atoms of mPEG. Furthermore, it has been reported that lysine and arginine residues can coordinate with PEG to form a CR-like structure, in which a cyclic PEG is wrapped as crown ether analogue^24,31^. The second semicircular CR-like structure is also wrapped around the guanidine group of R101_H,_ which forms a possible H-bond with the oxygen atoms of mPEG (Fig. 3a). Unsurprisingly, mutation of R101_H_ to alanine (R101_H_A) completely abolished mPEG-binding (Fig. 3d; Supplementary Table 1). We further generated a R101_H_K mutant to investigate whether lysine is able to interact with mPEG to form the CR-like structure. However, due to the crucial interaction between R101_H_ and Y96_L_ (Supplementary Fig. 3), the CR-like structure of mPEG mediated by R101_H_K mutant might be unstable, resulting in the elimination of the mPEG-binding (Fig. 3d; Supplementary Table 1). This result supports that the interaction between R101_H_ and Y96_L_ is important for mPEG binding and might stabilize the CR-like structure of mPEG.

### Characterization of important residues of h15-2b for mPEG-binding

Based on the crystal structure, four hydrophobic residues, V37_H_, L89_L_, F98_L,_ and W104_H,_ are close to the methoxy group of mPEG, thereby raising an important question, how h15-2b specifically binds to PEG molecules with a terminal methoxy group. Therefore, we generated L89_L_D, F98_L_E, F98_L_Y, W104_H_Y, W104_H_E V37_H_S, and V37_H_T Fab variants, in which the hydrophobic side chains were replaced by the polar side chains to test whether these mutants can switch their binding specificity from mPEG to OH-PEG or SH-PEG. None of the h15-2b variants bound to SH-PEG while L89_L_D, F98_L_E, and W104_H_E variants exhibited impaired binding to mPEG and OH-PEG (Supplementary Fig. 4; Fig. 4a; Supplementary Table 1). By contrast, F98_L_Y variant maintained similar mPEG-binding as compared to wild-type h15-2b but showed limited interaction with OH-PEG (Fig. 4a; Supplementary Table 1). Interestingly, mutating the hydrophobic W104_H_ and V37_H_ to a polar amino acid, tyrosine or serine, or threonine (W104_H_Y, V37_H_S, and V37_H_T) enhanced mPEG-binding and mildly increased binding to OH-PEG, possibly by formation of hydrogen bonds to methoxy and hydroxyl groups on the mPEG and OH-PEG molecules, respectively (Fig. 4b). Taken together, Fig. 4 reveals that hydrophilic amino acids (L89_L_D, F98_L_E, and W104_H_E) might reduce binding to mPEG and OH-PEG. On the other hand, all h15-2b variants are unable to bind OH-PEG, except for W104_H_Y, V37_H_S, and V37_H_T mutants, which mildly increase binding to OH-PEG, indicating that W104_H_ and V37_H_ is critical for specificity to mPEG (Supplementary Fig. 4; Supplementary Table 1).

## Discussion

We report the first crystal structure of an antibody that specifically binds PEG with a terminal methoxy group. Aromatic amino acids in both framework and CDR regions stabilize the hook-shaped CR-like structure of the PEG chain via van der Waals and H-bond interactions. Hydrophobic residues, including V37_H_, L89_L_, F98_L_, and W104_H_, provide the methoxy-specificity of h15-2b, which are also highly conserved throughout antibodies to maintain the stability of the V_H_:V_L_ interface^32^. Interestingly, V37_H_S and V37_H_T variants can partially cross-react with OH-PEG, indicating that V37_H_ is mostly important for the selectivity of h15-2b toward methoxy-terminated PEG.

Note that although V37_H_, L89_L_, F98_L_, and W104_H_ residues are responsible for the methoxy-specificity of h15-2b, these residues are highly conserved throughout anti-PEG and anti-mPEG antibodies (Supplementary Fig. 5). We found that h15-2b possesses shorter heavy chain complementarity-determining region 3 (HCDR3) but similar light chain CDR3 (LCDR3) as compared to 3.3, 2B5, and 6.3 (Supplementary Fig. 5). Structural comparison of the anti-PEG and anti-mPEG Fabs reveals that the h15-2b HCDR3 loop coupled with these conserved residues assist to form a tunnel-like structure for capturing the mPEG deep in the V_H_:V_L_ interface (Supplementary Fig. 6a). By contrast, the bulky HCDR3 loops of these anti-PEG Fabs hinder the tunnel-like structure formation and provide large surface area to interact with the backbone of PEG (Supplementary Fig. 6b–d). Therefore, this might explain the unique methoxy-specificity of h15-2b.

The crystal structures of several backbone-specific anti-PEG antibodies in complex with PEG have been determined. Unlike 3.3, 2B5 and 6.3 anti-PEG backbone-specific antibodies that dimerize while interacting with PEG^24,25^, h15-2b binds mPEG as a monomer. Briefly, the PEG chain forms a planar or spiral S-shaped CR-like configuration while bridging two Fabs in their antigen-binding sites to form 3.3, 2B5, and 6.3 Fab homodimers (Supplementary Fig. 7). These planar symmetric CR-like structures of PEG are bound by backbone-specific anti-PEG antibodies via aromatic residues and coordinate with water molecules to form H-bond with the ether oxygen atoms of PEG^24^. By contrast, the mPEG chain is clamped by aromatic amino acids in the paratope surface area of h15-2b to form the first CR-like structure and then bent into the V_H_:V_L_ interface to form a second CR-like structure (Supplementary Fig. 7). Indeed, CR is a cyclic version of linear PEG that exhibits a more stable configuration. Therefore, despite PEG polymer being highly flexible with variant conformations, the similar CR-like structures clamped by aromatic amino acids or the guanidinium moiety of arginine are observed in both backbone-specific anti-PEG antibodies and the methoxy-specific h15-2b anti-mPEG antibody.

mPEG is widely used in medical products to improve the solubility and circulation time of therapeutic proteins and nanoparticles. Therefore, both anti-PEG and anti-mPEG antibodies generated in patients might hinder the therapeutic efficacy and safety of PEGylated medicine. Although pre-existing anti-PEG antibodies are present in many naïve individuals, the incidence of pre-existing anti-mPEG antibodies remains unexplored. Most assays were developed for measuring anti-PEG antibodies in serum samples using direct ELISA for binding to immobilized mPEG^18,21^. However, these direct ELISA assays are unable to distinguish anti-PEG and anti-mPEG antibodies since coated mPEG could be detected by both. Alternatively, we hypothesize that anti-mPEG antibodies might be employed for competitive ELISA to measure pre-existing anti-mPEG antibodies^22^. Supposedly, serum samples containing pre-existing anti-mPEG but not anti-PEG antibodies can compete with mPEG-biotin captured by immobilized h15-2b antibodies to obtain a dose-response curve.

Engineered bispecific PEG-binding antibodies (PEG engagers) that simultaneously bind PEG molecules and tumor-associated antigens have also been developed for targeted delivery of mPEGylated nanomedicine in cancer therapies^33–38^. Intriguingly, the efficacy of PEG engager-directed mPEGylated nanomedicine is not compromised by pre-existing anti-PEG antibodies due to the high anti-PEG affinity of the PEG engagers^35^. Unlike chemical conjugation of targeting ligands with mPEGylated nanomedicine that require complicated covalent conjugation process, PEG engagers allow non-covalent mixing with any mPEGylated nanomedicine to reduce manufacturing complexity for targeted therapy. However, it has been reported that non-covalent antibody-payload complex dissociate in vivo after extended periods of time and presumably influence their effectiveness^39^. To further improve the in vivo long-term stability of PEG engager-directed nanomedicine, affinity improvement of anti-mPEG antibodies is required. In conclusion, the structural and functional analyses of h15-2b not only elucidate the mPEG-binding mechanism of anti-mPEG antibodies but also provide useful insights to assist in the rational design of high-affinity anti-mPEG antibodies. This might be accomplished by the combination of the W104_H_Y, V37_H_S and V37_H_T mutations to create high-affinity h15-2b variants for the analytical study of pre-existing anti-mPEG antibodies and the development of efficient targeted mPEG-nanomedicine.

## Methods

### Production of humanized anti-mPEG and anti-PEG Fabs

The V_L_-Cκ and V_H_-CH_1_ DNA fragments of anti-mPEG h15-2b or anti-PEG 6.3 were linked by a composite internal ribosome entry site bicistronic expression element and inserted into the pLPCX plasmid containing a C-terminal polyhistidine-tag to generate pLPCX-anti-mPEG Fab-6xHis and pLPCX-anti-PEG Fab-6xHis plasmids. ExpiCHO-S cells were transfected with pLPCX-anti-mPEG Fab-6xHis and pLPCX-anti-PEG Fab-6xHis using ExpiFectamine CHO Transfection Kit (Thermo Fisher Scientific, San Jose, CA) according to the manufacturer’s instructions. Culture supernatant was harvested 10 days post-transfection by centrifugation at 1000 x *g* for 5 min and then filtered through a 0.45 μm filter. The polyhistidine-tagged Fab was purified on a HiTrap TALON crude column (Cytiva, Marlborough, MA). Protein concentrations were determined by the Pierce BCA Protein Assay Kit (Thermo Fisher Scientific, San Jose, CA).

### Crystallization and data collection

Anti-mPEG h15-2b Fab was crystallized using the hanging-drop vapor diffusion method. The anti-mPEG Fab was buffer exchanged in 20 mmol L^−1^ Tris containing 100 mmol L^−1^ NaCl, pH 7.5, and concentrated to 21 mg mL^−1^. The complex crystals of the anti-mPEG Fab/mPEG were grown by mixing 1 μL protein solution (21 mg mL^−1^) with 1 μL reservoir solution using the sitting-drop vapor diffusion method at 20 °C. The anti-mPEG Fab/mPEG crystals were crystallized under a screen condition: 18% (w/v) PEG-6000, 1% (w/v) PEG-2000 methyl ether, 0.15 mol L^−1^ lithium sulfate monohydrate, and 0.1 mol L^−1^ citric acid (pH 3.5) at 20 °C (Sigma–Aldrich, St. Louis, MO). Snap-freezing the crystals with 20% glycerol (v/v) as a cryoprotectant for X-ray data collection at cryogenic temperatures (80 K). The diffraction data of anti-mPEG Fab/mPEG crystals were collected at the National Synchrotron Radiation Research Center (NSRRC, Taiwan) on beamline BL13B1 using a wavelength of 0.9732 Å with the ADSC QUANTUM 315r CCD detector (Area Detector Systems Corporation, Poway, CA). Data were indexed and scaled using HKL2000.

### Structure determination and refinement

The crystal structure of anti-mPEG Fab in complex with mPEG was determined by the molecular replacement method of MOLREP using the structure of the anti-ErbB2 Fab2C4 (Protein Data Bank entry 1L7I) as a search model^29^. Extensive manual model building and refinement were performed using COOT^40^. The models were further refined with REFMAC^41^. The final model shows 0.48% outliers and 95.04% of residues in the most favored regions and 4.48% of residues in the additionally allowed regions of the Ramachandran diagram. The atomic coordinates and structure factors of h15-2b Fab/mPEG were deposited in the Protein Data Bank as entry 7Y0G. PyMol was used to generate figures of structures.

### Generation of h15-2b Fab variants by site-directed mutagenesis

The wild-type light chain and heavy chain with a C-terminal polyhistidine-tag of h15-2b Fab were cloned into a dual-expression plasmid containing the pelB and the stII signal peptides for periplasm expression in Escherichia coli. The parental h15-2b Fab plasmid was employed as a template to amplify h15-2b V_H_ and V_L_ mutant fragments by site-directed mutagenesis using overlap extension PCR^30^. The parental plasmids were linearized by digesting with BsiwI/NheI or NcoI/RsrII (New England Biolabs, Ipswich, MA) to remove original V_H_ or V_L_ fragments, respectively. h15-2b mutant amplicons were cloned into the linearized vector using Gibson Assembly cloning. Each of the h15-2b Fab variants was transformed into *E. coli* C43 (DE3). The resultant recombinant strains were culture in 2xYT Broth at 37 °C and induced with 1 mmol L^−1^ of isopropyl β-D-1-thiogalactopyranoside (IPTG) (Sigma–Aldrich, St. Louis, MO) when an OD_600_ of 0.5 was reached. The cells were incubated at 30 °C for 20 h after IPTG induction. The cells were collected by centrifugation at 3000 x *g* for 15 min at 4 °C, and the pellet was resuspended in lysis buffer (100 mmol L^−1^ Tris, 300 mmol L^−1^ NaCl, 1 mmol L^−1^ phenylmethanesulfonyl fluoride, pH 8.0) (Sigma–Aldrich, St. Louis, MO). After sonication, the clear lysate was harvested by centrifugation at 12,000 x *g* for 15 min at 4 °C and filtered through a 0.45 μm filter. Each polyhistidine-tagged h15-2b mutant Fabs was purified on a HiTrap TALON crude column (Cytiva, Marlborough, MA) and buffer exchanged in PBS buffer for further characterization.

### Antibody ELISA

Maxisorp 96-well microplates (Thermo Fisher Scientific, San Jose, CA) were coated with 0.5 µg per well of mPEG_5K_-NH_2_ or OH-PEG_5K_-NH_2_ (Nanocs, New York, NY) or SH-PEG_3.5K_-NH_2_ (Sigma–Aldrich, St. Louis, MO) or recombinant GFP (Cell Biolabs, Inc., San Diego, CA) in 50 µL 100 mmol L^−1^ NaHCO_3_/Na_2_CO_3_ coating buffer (pH 8.0) for 3 h at 37 °C and then blocked with 200 µL of 5% (wt/vol) skim milk in PBS at 4 °C overnight. Graded concentrations of purified h15-2b Fab variants or 6.3 Fab or anti-GFP Fab in 50 µL 2% (wt/vol) skim milk was added to the plates at RT for 1 h. The plates were washed with PBS three times. HRP-conjugated goat anti-human F(ab’)_2_ fragment specific antibodies (1 µg mL^−1^) (Jackson Immuno Research Laboratories, West Grove, PA, catalog number: 109-035-097) in 50 µL 2% (wt/vol) skim milk was added for 30 min at room temperature. The plates were washed with PBS three times, and bound peroxidase activity was measured by adding 150 µL per well of ABTS substrate solution (0.4 mg mL^−1^ 2,2’-azino-di (3-ethylbenzthiazoline-6-sulfonic acid) (Sigma–Aldrich, St. Louis, MO), 0.003% H_2_O_2_, 100 mmol L^−1^ phosphate citrate, pH 4.0) for 30 min at room temperature. The absorbance (405 nm) was measured in a SpectraMax ABS Plus microplate reader (Molecular Device, Menlo Park, CA). GraphPad Prism 6 was used to analyze ELISA data. The anti-GFP Fab was kindly provided by Dr. Kurt Yun Mou (Academia Sinica, Taipei, Taiwan). Note that both Maxisorp (Thermo Fisher Scientific, San Jose, CA) and Well-Coated™ Amine Binding microplates (G-Biosciences, St. Louis, MO) are widely used for immobilization of amine group containing antigens. Comparison of these microplates for anti-PEG ELISA revealed that there is no difference between them (Supplementary Fig. 8). Therefore, the Maxisorp microplates were used for all ELISA experiments in this study.

### Reporting summary

Further information on research design is available in the Nature Research Reporting Summary linked to this article.

### Supplementary information

Su_PR File

Supplementary Information

Description of Additional Supplementary Files

Supplementary Data 1

Reporting Summary

## Data Availability

The data that support the findings of this study are available from the corresponding author on reasonable request. The Protein Data Bank data of h15-2b Fab/mPEG crystal structure can be found in Supplementary Data 1. The atomic coordinates and structure factors of h15-2b Fab/mPEG were deposited in the Protein Data Bank as entry 7Y0G.
