# Supplementary material for: Structural determination of an antibody that specifically recognizes polyethylene glycol with a terminal methoxy group
Source: Commun Chem. 2022 Aug 1;5:88. doi: 10.1038/s42004-022-00709-0 (PMC9340711; doi:10.1038/s42004-022-00709-0)
Supplement: Supplementary file 2 — Supplementary Information [file 42004_2022_709_MOESM2_ESM.docx]

**Supplementary Information**

**Structural determination of an antibody that specifically recognizes polyethylene glycol with a terminal methoxy group**

Minh-Tram T. Nguyen^1#^, Yu-Chien Shih^1#^, Meng-Hsuan Lin^1#^, Steve R. Roffler^2^, Chiao-Yu Hsiao^1^, Tian-Lu Cheng^3^, Wen-Wei Lin^4^, En-Chi Lin^1^, Yuh-Jyh Jong^5^, Chin-Yuan Chang^1, 3*^ and Yu-Cheng Su^1, 3*^

^1^Department of Biological Science and Technology, Center for Intelligent Drug Systems and Smart Bio-devices (IDS^2^B), National Yang Ming Chiao Tung University, Hsinchu, Taiwan. ^2^Institute of Biomedical Sciences, Academia Sinica, Taipei, Taiwan. ^3^Department of Biomedical Science and Environmental Biology, Drug Development and Value Creation Research Center, Kaohsiung Medical University, Kaohsiung, Taiwan. ^4^School of Post-Baccalaureate Medicine, College of Medicine, Kaohsiung Medical University, Kaohsiung, Taiwan. ^5^Graduate Institute of Clinical Medicine, Departments of Pediatrics and Laboratory Medicine, and Translational Research Center of Neuromuscular Diseases, Kaohsiung Medical University, Kaohsiung, Taiwan.

# These authors contributed equally:

Minh-Tram T. Nguyen, Yu-Chien Shih and Meng-Hsuan Lin

*Corresponding authors:

Chin-Yuan Chang, cycytl@nycu.edu.tw or Yu-Cheng Su, ycsu-johnny@nctu.edu.tw

**Supplementary Methods**

**Competitive ELISA**

Maxisorp 96-well microplates (Thermo Fisher Scientific, San Jose, CA) were coated with 2 µmol L^-1^ of mPEG_2K_-NH_2_ (Nanocs, New York, NY) or recombinant TNF-α (PeproTech, Cranbury, NJ) in 50 µL 100 mmol L^-1^ NaHCO_3_/Na_2_CO_3_ coating buffer (pH 8.0) for 3 h at 37 °C and then blocked with 200 µL of 5% (wt/vol) skim milk in PBS at 4 °C overnight. A fixed concentration of Fab (0.5 µmol L^-1^) was mixed at a 1:1 volume ratio with graded concentrations (0.01–24 µmol L^-1^) of OH-PEG_6K_ or mPEG_5K_ (Sigma-Aldrich, St. Louis, MO) in 50 µL 2% (wt/vol) skim milk and then added to the plates for 1 h at room temperature. The plates were washed with PBS three times. HRP-conjugated goat anti-human F(ab')_2_ fragment specific antibodies (1 µg mL^-1^) (Jackson Immuno Research Laboratories, West Grove, PA) in 50 µL 2% (wt/vol) skim milk were added for 30 min at room temperature. The plates were washed with PBS three times, and bound peroxidase activity was measured by adding 150 µL per well of ABTS substrate solution (0.4 mg mL^-1^ 2,2’-azino-di (3-ethylbenzthiazoline-6-sulfonic acid) (Sigma-Aldrich, St. Louis, MO), 0.003% H_2_O_2_, 100 mmol L^-1^ phosphate citrate, pH 4.0) for 30 min at room temperature. The absorbance (405 nm) was measured in a SpectraMax ABS Plus microplate reader (Molecular Device, Menlo Park, CA). Humira anti-TNF-α Fab was purchased from Rockland Immunochemicals (Pottstown, PA).

**Amino acid sequence alignment of variable domains**

The amino acid sequence alignment of variable domains of anti-PEG and anti-mPEG antibodies were compared by using Clustal Omega. Residues are numbered according to IMGT numbering and complementarity-determining regions CDRs were identified using the AbRSA online tool.


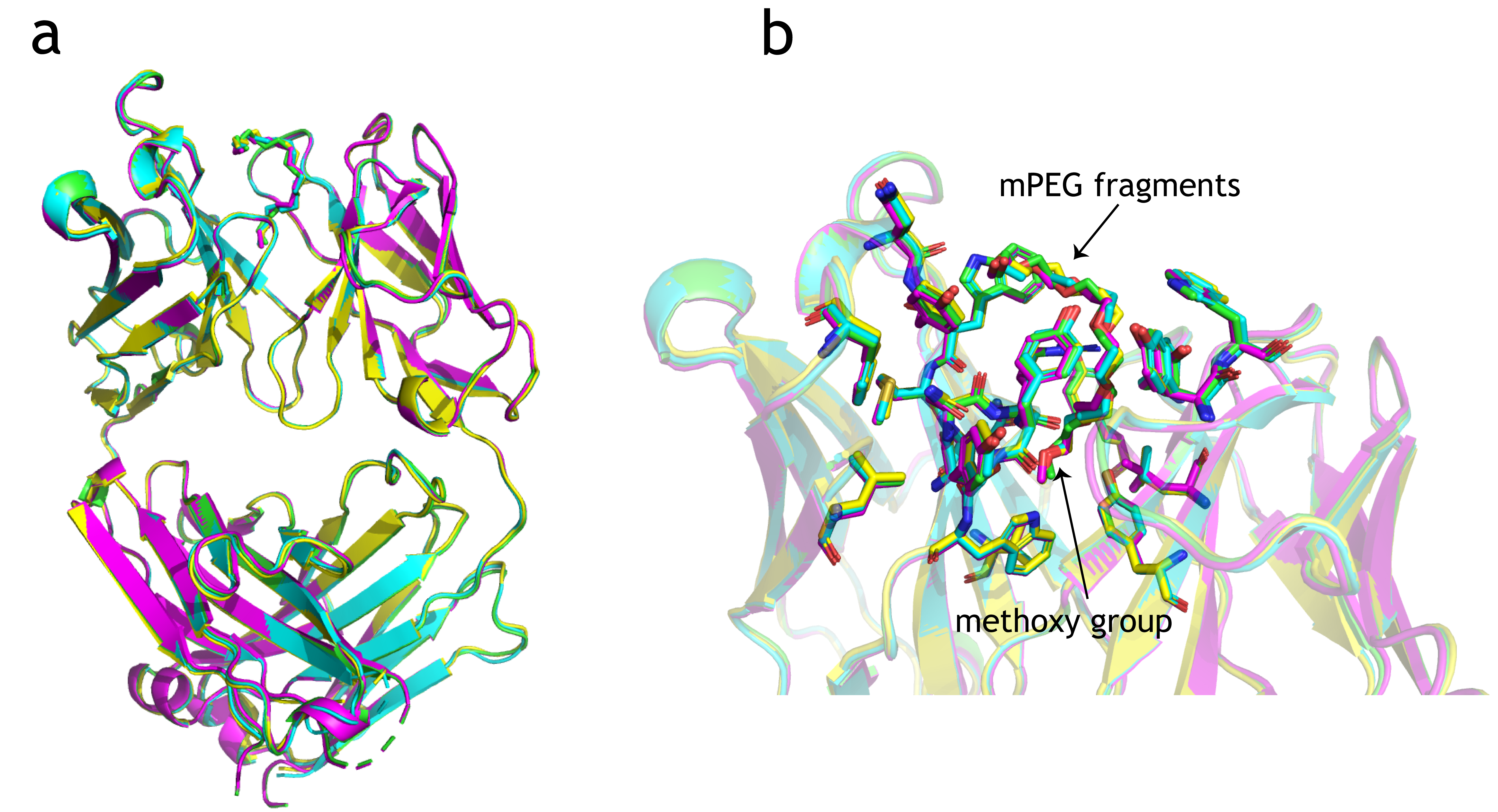


**Supplementary Fig. 1: The crystal structures of the four copies of h15-2b Fabs**. **a** Superposition of the overall structures of the four copies of h15-2b Fabs. **b** Local view of each mPEG binding site in the four copies of h15-2b Fabs. The four copies of h15-2b and mPEG fragments are colored green, cyan, magentas, and yellow, respectively.


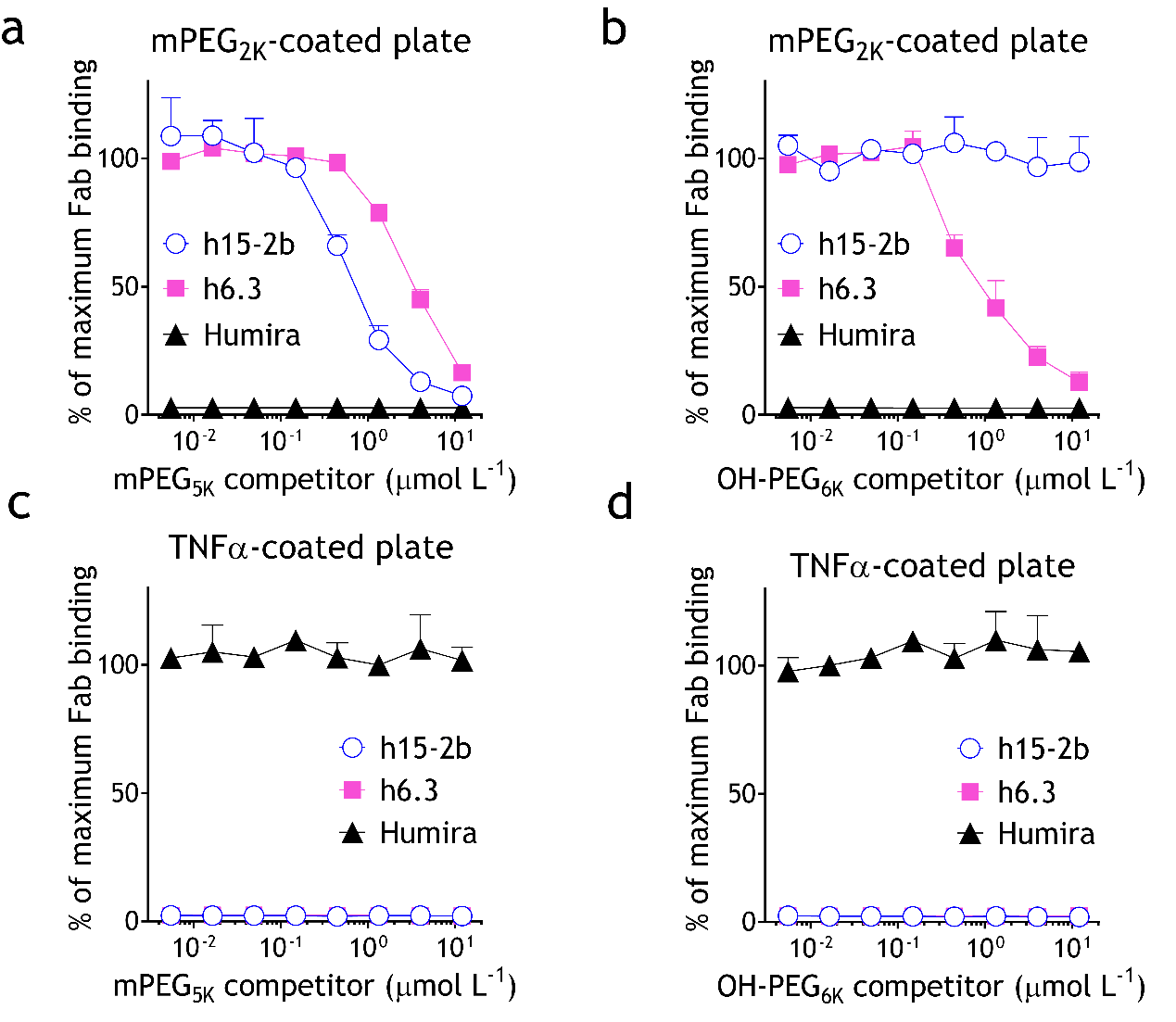
**Supplementary Fig. 2: The influence of excess OH-PEG in the anti-mPEG ELISA**. Graded concentrations of competitors (OH-PEG_6K_ or mPEG_5K_) were mixed with 0.5 µmol L^-1^ of h15-2b (open circle), h6.3 (closed square) or Humira (closed triangle) Fabs prior to addition to microplates coated with (**a, b**) mPEG_2K_-NH_2_ or (**c, d**) TNF-α. After washing, the Fab binding was determined by adding HRP-conjugated goat anti-human F(ab')_2_ fragment specific antibodies, followed by ABTS substrate. Results of triplicate determinations (n=3) show Fab binding as a percentage of maximum binding activity. Data are shown as mean ± standard deviation. The data are representative of three independent experiments.


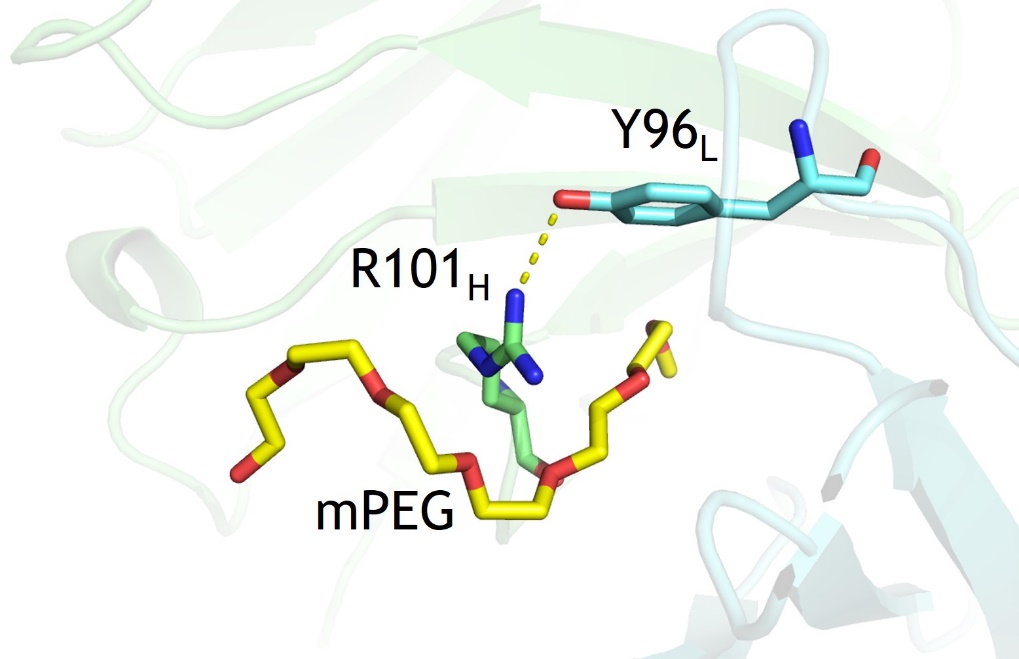
**Supplementary Fig. 3: The mPEG adopting a crown ether-like conformation to the side chain of R101_H_ in h15-2b Fab.** Close view of R101_H_ interaction with Y96_L_ and mPEG fragment. The mPEG forms a crown ether-like configuration with R101_H_ residue. The orientation of R101_H_ is stabilized by Y96_L_ residue via hydrogen bond interaction (yellow dash).


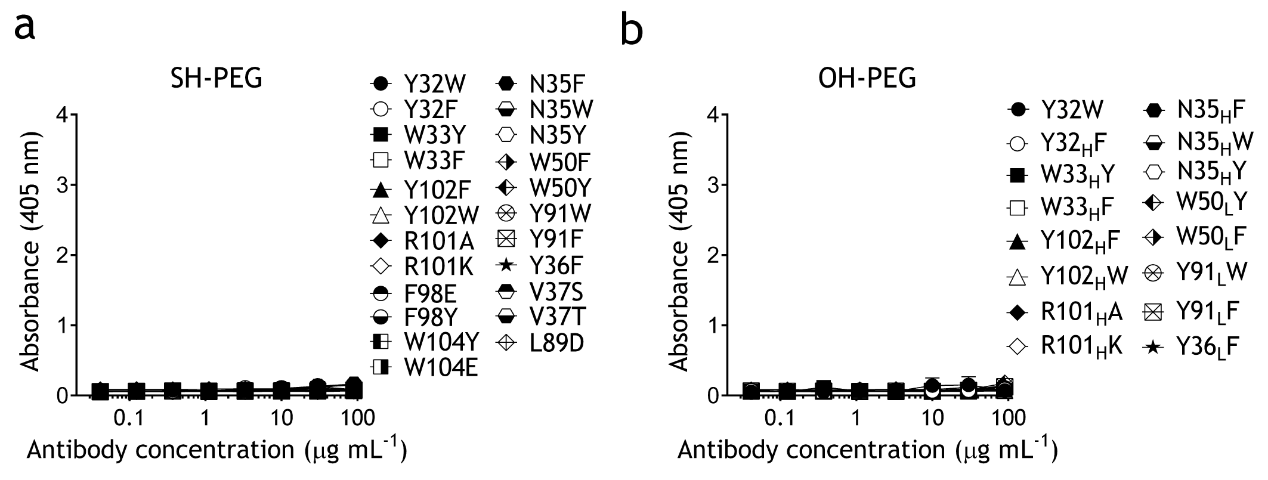
**Supplementary Fig. 4: Specificity of h15-2b Fab variants against thiol-PEG and hydroxyl-PEG molecules.** Microplate wells coated with (**a**) SH-PEG_3.5K_-NH_2_ or (**b**) OH-PEG_5K_-NH_2_ were incubated with graded concentrations of h15-2b variants. After 1h, the wells were washed, and antibody binding was determined by adding HRP-conjugated goat anti-human F(ab')_2_ fragment specific antibodies, followed by ABTS substrate. The results show the mean absorbance values (405 nm) ± standard deviation (n=3). The data are representative of three independent experiments.

**
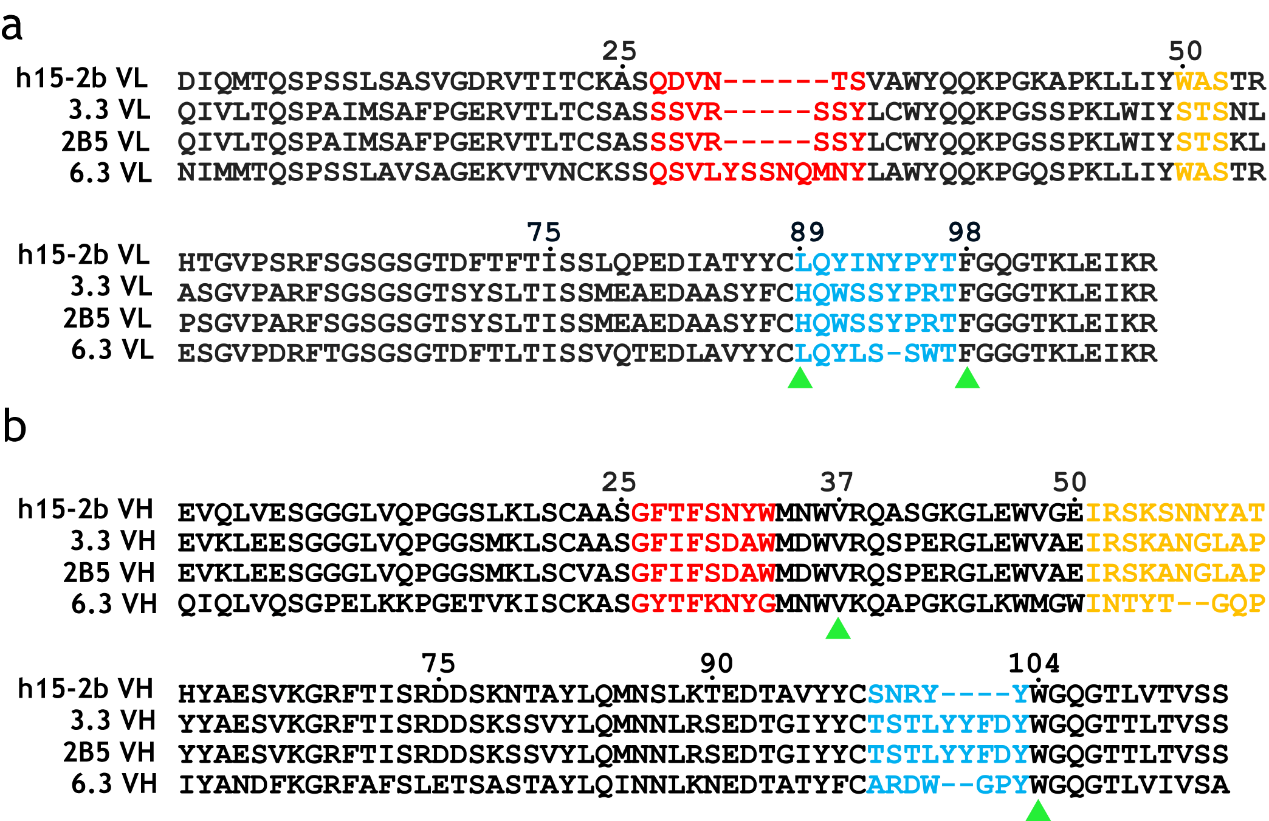
Supplementary Fig. 5: Variable domain amino acid sequence alignments of anti-PEG and anti-mPEG antibodies**. Amino acid sequence alignments of (**a**) variable light chain domain (V_L_) and (**b**) Variable heavy chain domain (V_H_) for anti-mPEG (h15-2b) and anti-PEG (3.3, 2B5 and 6.3) antibodies. The CDR regions are highlighted (CDR1, red; CDR2, yellow; CDR3, blue) and the conserved residues, including L89_L_/H89_L_, F98_L_, V37_H_ and W104_H_ are indicated with green triangles.


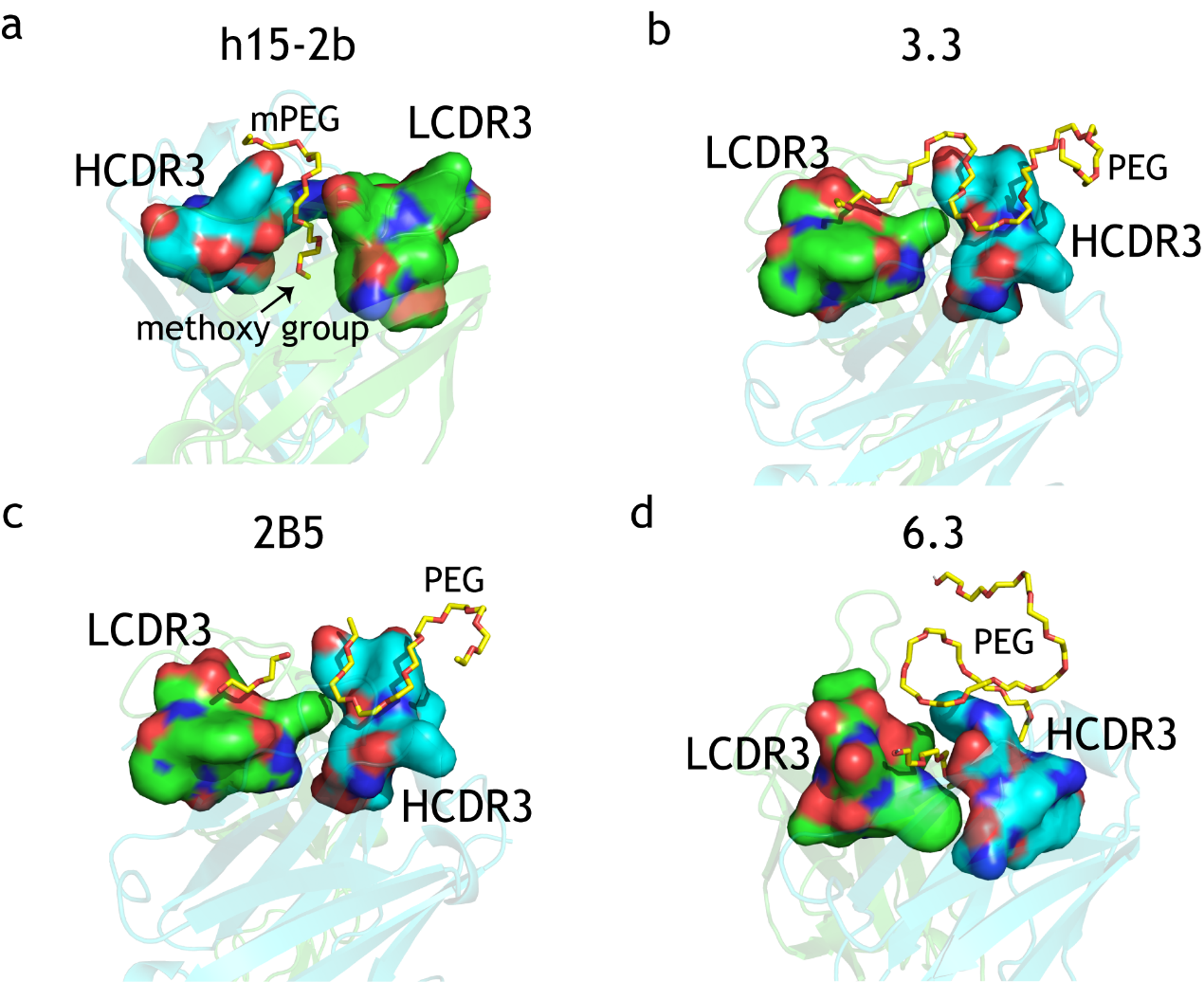
**Supplementary Fig. 6: Structural comparison of anti-mPEG and anti-PEG antibodies**. **a** h15-2b; **b** 3.3; **c** 2B5; **d** 6.3. Surface representation of HCDR3 and LCDR3 are colored in cyan and green, respectively. Oxygen and nitrogen are colored in red and blue, respectively in the surface representation of CDRs. mPEG or PEG are colored yellow. The methoxy group of a mPEG is indicated with an arrow.


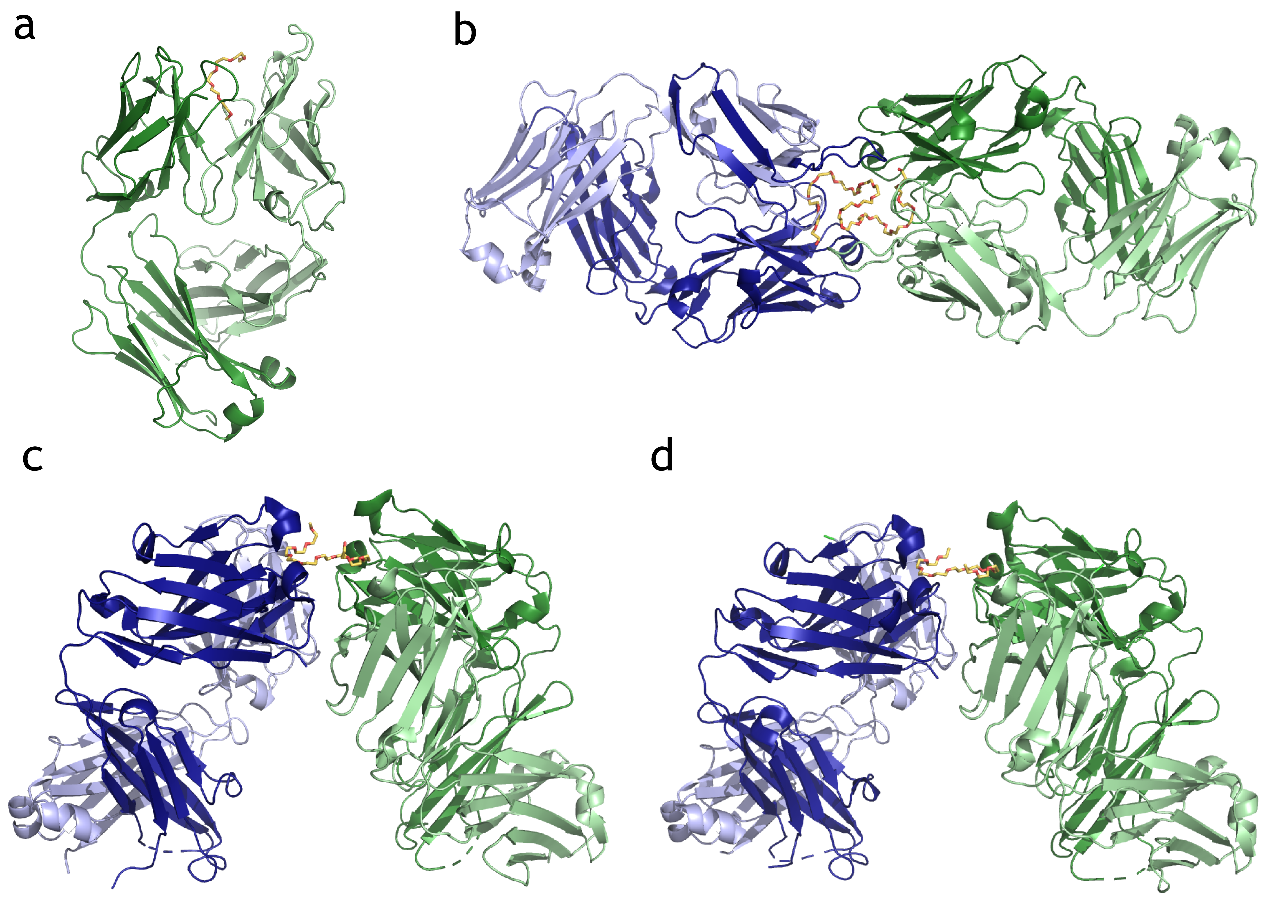
**Supplementary Fig. 7: The crystal structures of anti-mPEG and anti-PEG antibodies.** **a** h15-2b; **b** 6.3; **c** 3.3; **d** 2B5. The two Fab subunits are colored green and blue. The heavy chain and light chain of Fab are colored in deep and light color, respectively. mPEG or PEG are colored yellow.

**Supplementary Fig. 8: Comparison of maxisorp and amine-binding plates for anti-PEG ELISA.
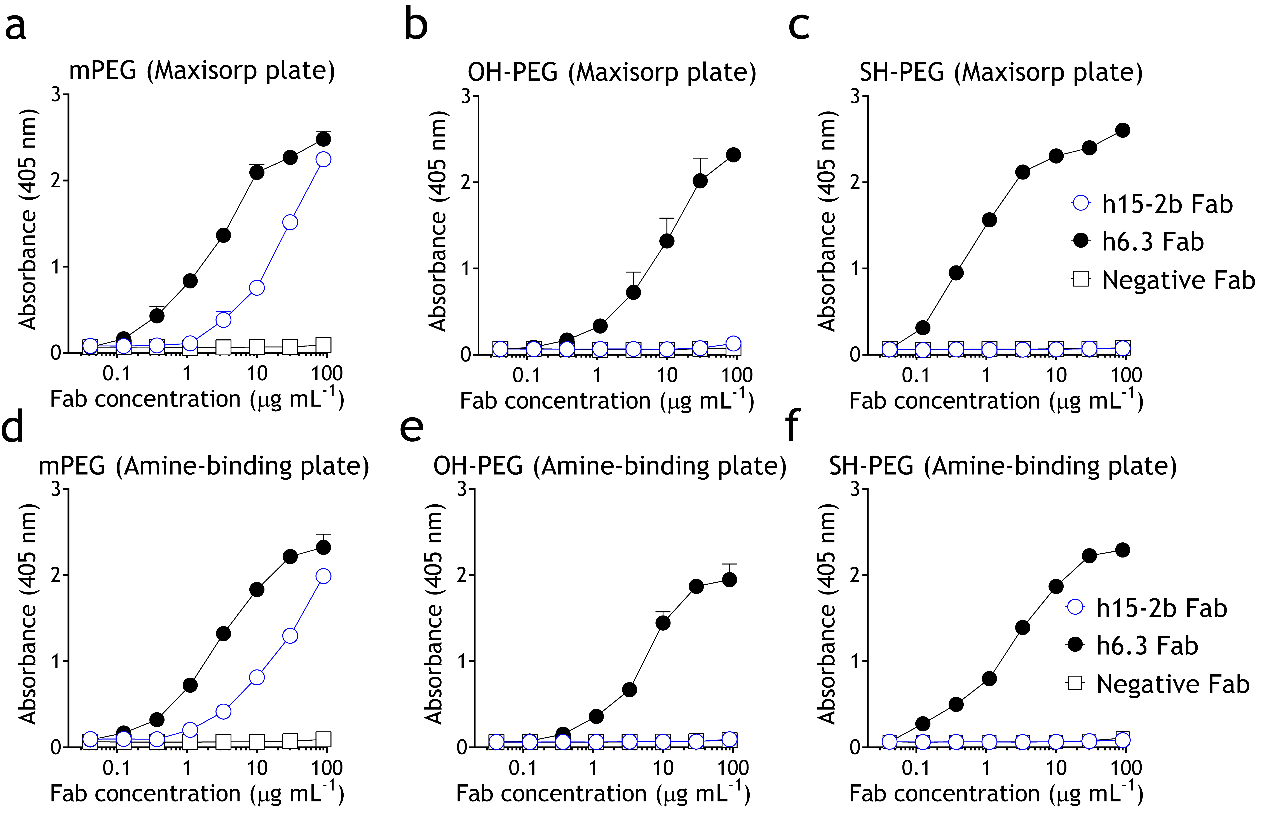
**Maxisorp (**a-c**) or amine-binding (**d-f**) microplate wells coated with amino-PEG molecules terminated (**a, d**) methoxy, (**b, e**) hydroxyl, or (**c, f**) thiol groups were incubated with graded concentrations of methoxy-specific (h15-2b, open circle) or backbone-specific (h6.3, closed circle) anti-PEG Fabs or anti-GFP Fab (negative control, open square). After washing, the Fab binding was determined by adding HRP-conjugated goat anti-human F(ab')_2_ fragment specific antibodies, followed by ABTS substrate. The results show the mean absorbance values (405 nm) ± standard deviation (n=3). The data are representative of three independent experiments.

**
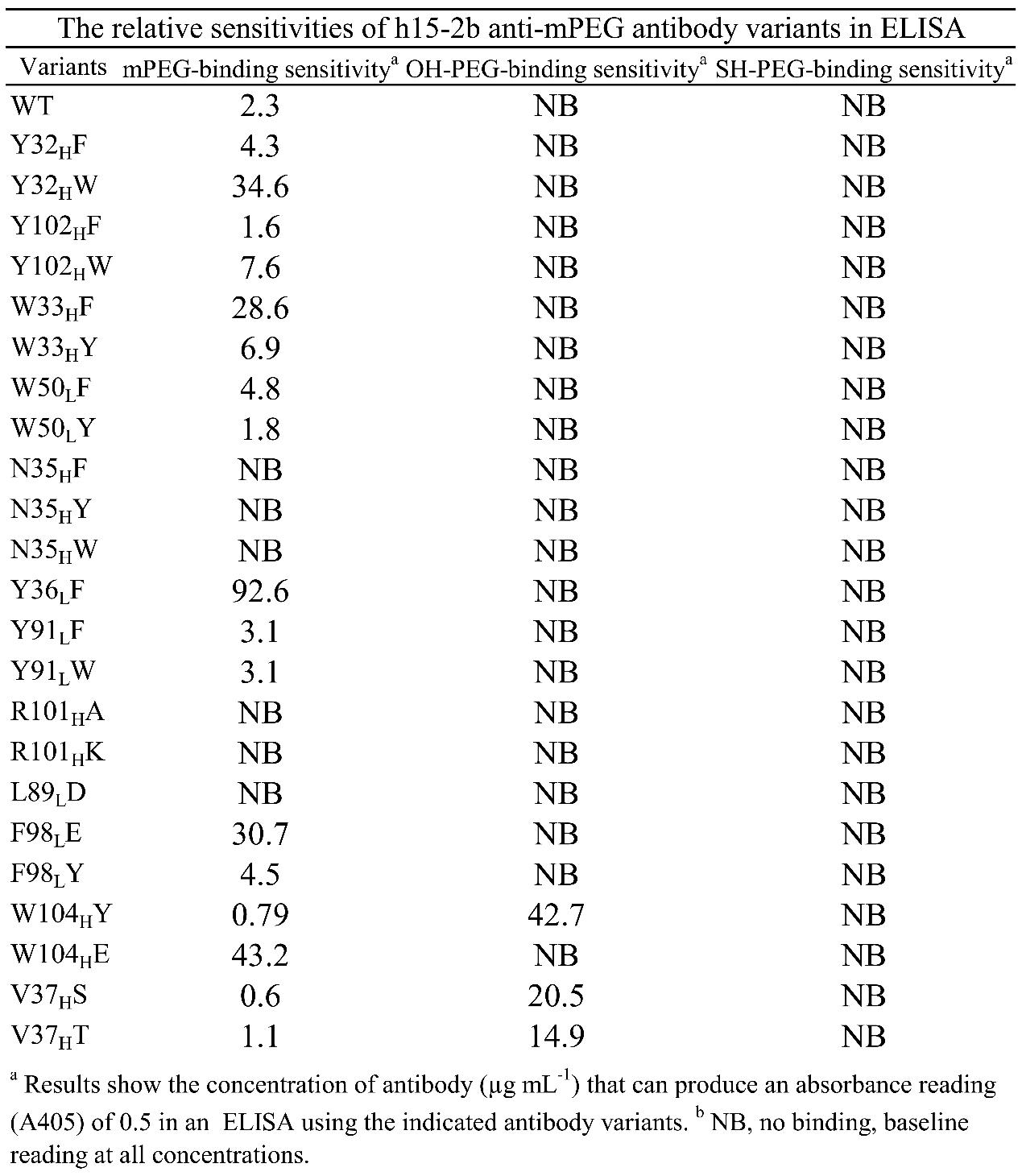
Supplementary Table 1: The relative sensitives of anti-mPEG antibody variants.**
