# Supplementary material for: Structural determination of an antibody that specifically recognizes polyethylene glycol with a terminal methoxy group
Source: Commun Chem. 2022 Aug 1;5:88. doi: 10.1038/s42004-022-00709-0 (PMC9340711; doi:10.1038/s42004-022-00709-0)
Supplement: Supplementary file 3 — Description of Additional Supplementary Files [file 42004_2022_709_MOESM3_ESM.docx]

Description of Additional Supplementary Files

**File name:** Supplementary Data 1

**Description:** The Protein Data Bank data of h15-2b Fab/mPEG crystal structure
