# Supplementary material for: Structural determination of an antibody that specifically recognizes polyethylene glycol with a terminal methoxy group
Source: Commun Chem. 2022 Aug 1;5:88. doi: 10.1038/s42004-022-00709-0 (PMC9340711; doi:10.1038/s42004-022-00709-0)
Supplement: Supplementary file 5 — Reporting Summary [file 42004_2022_709_MOESM5_ESM.pdf]

nature research

Corresponding author(s): Yu-Cheng Su

Last updated by author(s): 2022/07/09

Reporting Summary

Nature Research wishes to improve the reproducibility of the work that we publish. This form provides structure for consistency and transparency in reporting. For further information on Nature Research policies, see our Editorial Policies and the Editorial Policy Checklist.

Statistics

For all statistical analyses, confirm that the following items are present in the figure legend, table legend, main text, or Methods section.

n/a	Confirmed	
☐	☒	The exact sample size (n) for each experimental group/condition, given as a discrete number and unit of measurement
☐	☒	A statement on whether measurements were taken from distinct samples or whether the same sample was measured repeatedly
☒	☐	The statistical test(s) used AND whether they are one- or two-sided<br>*Only common tests should be described solely by name; describe more complex techniques in the Methods section.*
☒	☐	A description of all covariates tested
☒	☐	A description of any assumptions or corrections, such as tests of normality and adjustment for multiple comparisons
☐	☒	A full description of the statistical parameters including central tendency (e.g. means) or other basic estimates (e.g. regression coefficient) AND variation (e.g. standard deviation) or associated estimates of uncertainty (e.g. confidence intervals)
☒	☐	For null hypothesis testing, the test statistic (e.g. F, t, r) with confidence intervals, effect sizes, degrees of freedom and P value noted<br>*Give P values as exact values whenever suitable.*
☒	☐	For Bayesian analysis, information on the choice of priors and Markov chain Monte Carlo settings
☒	☐	For hierarchical and complex designs, identification of the appropriate level for tests and full reporting of outcomes
☒	☐	Estimates of effect sizes (e.g. Cohen's d, Pearson's r), indicating how they were calculated

Our web collection on statistics for biologists contains articles on many of the points above.

Software and code

Policy information about availability of computer code

Data collection	ELISA: SpectraMax ABS Plus microplate reader (Molecular Device, Menlo Park, CA)<br>Diffraction data: ADSC QUANTUM 315r CCD detector (Area Detector Systems Corporation, Poway, CA)
Data analysis	GraphPad Prism 6 was used to analyze ELISA data.<br>PyMol was used to generate figures of structures

For manuscripts utilizing custom algorithms or software that are central to the research but not yet described in published literature, software must be made available to editors and reviewers. We strongly encourage code deposition in a community repository (e.g. GitHub). See the Nature Research guidelines for submitting code & software for further information.

Data

Policy information about availability of data

All manuscripts must include a data availability statement. This statement should provide the following information, where applicable:

- Accession codes, unique identifiers, or web links for publicly available datasets
- A list of figures that have associated raw data
- A description of any restrictions on data availability

The data that support the findings of this study are available from the corresponding author on reasonable request.

Field-specific reporting

Please select the one below that is the best fit for your research. If you are not sure, read the appropriate sections before making your selection.

☒ Life sciences ☐ Behavioural & social sciences ☐ Ecological, evolutionary & environmental sciences

For a reference copy of the document with all sections, see nature.com/documents/nr-reporting-summary-flat.pdf

Life sciences study design

All studies must disclose on these points even when the disclosure is negative.

Sample size	No sample size calculation was performed. Triplicate determinations were performed in ELISA experiments, as this is the minimum number for statistical testing.
Data exclusions	No data was excluded.
Replication	Three independent experiments are performed in ELISA experiments. All replicate experiments of antigen-binding activity for antibodies by ELISA were successful.
Randomization	No randomization was performed. All antibody variants were analyzed for the PEG-binding activities in the same experimental settings.
Blinding	Blinding was not relevant to this study because there is no bias for studying PEG-binding activities of anti-mPEG antibody variants by ELISA.

Reporting for specific materials, systems and methods

We require information from authors about some types of materials, experimental systems and methods used in many studies. Here, indicate whether each material, system or method listed is relevant to your study. If you are not sure if a list item applies to your research, read the appropriate section before selecting a response.

Materials & experimental systems

n/a	Involved in the study	
☐	☒	Antibodies
☐	☒	Eukaryotic cell lines
☒	☐	Palaeontology and archaeology
☒	☐	Animals and other organisms
☒	☐	Human research participants
☒	☐	Clinical data
☒	☐	Dual use research of concern

Methods

n/a	Involved in the study	
☒	☐	ChIP-seq
☒	☐	Flow cytometry
☒	☐	MRI-based neuroimaging

Antibodies

Antibodies used	The secondary antibody for ELISA: HRP-conjugated goat anti-human F(ab')2 fragment specific antibodies, Jackson Immuno Research Laboratories, West Grove, PA; catalog number: 109-035-097; 1 μg/mL
Validation	The secondary antibody for ELISA:<br>HRP-conjugated goat anti-human F(ab')2 fragment specific antibodies, Jackson Immuno Research Laboratories, 109-035-097.<br>Specificity: IgG, $F(ab')_2$ fragment specific.<br>Minimal Cross Reactivity: Bovine, Horse, Mouse Serum Proteins.<br>Clonality: Polyclonal

Eukaryotic cell lines

Policy information about cell lines

Cell line source(s)	ExpiCHO
Authentication	None of the cell lines used were authenticated
Mycoplasma contamination	Cell lines used were not tested for mycoplasma contamination
Commonly misidentified lines (See ICLAC register)	Commonly misidentified lines were NOT used in this study
