# Supplementary material for: Structural determination of an antibody that specifically recognizes polyethylene glycol with a terminal methoxy group
Source: Commun Chem. 2022 Aug 1;5:88. doi: 10.1038/s42004-022-00709-0 (PMC9340711; doi:10.1038/s42004-022-00709-0)
Supplement: Supplementary file 1 — Su_PR File [file 42004_2022_709_MOESM1_ESM.pdf]

nature portfolio

Peer Review File

Structural determination of an antibody that specifically recognizes polyethylene glycol with a terminal methoxy group

Open Access This file is licensed under a Creative Commons Attribution 4.0 International License, which permits use, sharing, adaptation, distribution and reproduction in any medium or format, as long as you give appropriate credit to the original author(s) and the source, provide a link to the Creative Commons license, and indicate if changes were made. In the cases where the authors are anonymous, such as is the case for the reports of anonymous peer reviewers, author attribution should be to 'Anonymous Referee' followed by a clear attribution to the source work. The images or other third party material in this file are included in the article's Creative Commons license, unless indicated otherwise in a credit line to the material. If material is not included in the article's Creative Commons license and your intended use is not permitted by statutory regulation or exceeds the permitted use, you will need to obtain permission directly from the copyright holder. To view a copy of this license, visit http://creativecommons.org/licenses/by/4.0/.

Reviewers' comments:

Reviewer #1 (Remarks to the Author):

COMMSCHEM-22-0091
Title: Structural determination of an antibody that specifically recognizes polyethylene glycol with a terminal methoxy group.
In this research article, the authors build on previous work of elucidating the crystal structures of PEG-specific antibodies. In this article, they focus on antibody interactions with methoxy-PEG, using Fab molecules that are specific for the terminal group. The topic should be of interest to the community working on anti-PEG antibodies, and I found the mutagenesis data particularly interesting. I believe the work will be valuable for researchers trying to understand the nature of the interactions between antibodies and PEG. I suggest publication after minor revisions.

Specific comments:
I am not an expert in protein crystallization. However, I noticed that the authors crystallized the proteins in 18% OH-PEG-OH solutions (in the presence of 1% mPEG). I believe the authors should comment on the possibility of non-specific interactions between the protein and the OH-PEG-OH from the crystallization solvent. In other words, what precautions were taken to ensure that the structure computed from the X-ray crystallography experiment truly represents specific interactions with mPEG, and not serendipitous interactions with the (more abundant) PEG from the crystallization media? In previous published work, when the epitope was the backbone, this consideration might have been of lesser importance (the crystallization media would have the same backbone) – but to understand interactions with mPEG specifically, I believe clarifications might be in order.

I suggest the authors find a way of making the names of Fab variants more uniform. In some places (including figures), names are provided by using both the WT and mutant amino acids (Y91LW). Elsewhere, the mutants are referred to as "mutation of R100H to alanine". I believe reference between text and figures might be facilitated if the labelling was more uniform.

L87- The authors write: "PEG used in all therapeutic medicines is terminated by a methoxy group" – this statement should be revised: pegcetacoplan (Apellis pharma) is a pegylated peptide without a methoxy group, many drugs contain surfactants (notably polysorbate) that are not methoxy-terminated.

I suggest the authors find a way of identifying the methoxy group in FIG2. Currently, the representation of the mPEG molecule has a carbon at both ends, which makes it unclear to fully understand where the end-group is.

L226 – In the sentence starting with "these results suggest…", it is unclear to me what 'these results' refer to.

In the discussion, the authors summarize the hydrophobic residues that are important for mPEG binding (L267). It would be very interesting if the authors could compare what amino acids are present at these positions, on other antibodies that are backbone specific (i.e., not mPEG-specific).

L271 – the authors write 'V37H plays an important role in 15-2b binding to methoxy terminated PEG'. From my understanding of the results, it seems like this amino acid is mostly important for selectivity toward methoxy-PEG (since mutants also seem to have higher affinity toward mPEG, In FIG4). I suggest correcting the sentence.

Minor comments;
Legend of FIG 1C is SH-PEG3.5k-NH2 – the method section suggests PEG3.4k was used.
L343 – The abbreviation PEG-2000-MME does not appear to be explained anywhere.

L354 – PDB abbreviation should be explained.

Reviewer #2 (Remarks to the Author):

The manuscript by Nguyen M.T. et al describes structural aspects of the interaction between anti-mPEG antibody and its putative antigen - mPEG. This study is of high value for the field of nanomedicine because mPEG is widely used in the preparation of nanocarriers used for drug delivery. The study is also of value for the therapeutic proteins field because mPEG is widely used to improve protein therapeutics' solubility and circulation time. One of the original goals of using mPEG in both nanomedicine and therapeutic proteins was to create a hydrophilic shield around nanoparticles or proteins to shield them from the immune system and prevent clearance by the MPS, also to decrease the immunogenicity of recombinant proteins. However, multiple studies have demonstrated that PEG and mPEG are immunogenic and their use in protein therapeutics and nanoparticles is associated with the formation of anti-PEG antibodies. Moreover, exposure to PEG early in life result is anti-PEG antibodies in the blood of healthy individuals. The antibodies against PEG contribute to hypersensitivity reactions and affect both pharmacokinetics and clearance of PEGylated products. However, a fundamental understanding of antigenicity - the binding of anti-PEG antibodies and their antigen (PEG) - is missing in the literature. This study fills this gap. I recommend it for publication after revision to address several points which I summarize below:

1. For the ELISA assay, the authors use Maxisorp plates and bi-functional PEG containing NH2-terminal groups on one end and mPEG terminal groups on another end. This approach may not provide optimal conditions for the orientation of mPEG on the plate, with some PEG molecules having a theoretical chance to be flat or bind to the plate exposing NH2-terminus. Did the authors try using plates with a specialized surface for binding amines? For example, https://cdn.gbiosciences.com/pdfs/protocol/Well-Coated_Amine_Binding_Plates.pdf If there was no difference between these plates and maxisorp plate, it may be stated in the materials section for clarification.
2. Page 2, line 49 – I recommend removing "and immunogenicity of anti-PEG antibodies" because the study does not investigate PEG immunogenicity. The study details structural aspects of the interaction between the antibody (anti-PEG antibody) and antigen (PEG molecule). Alternatively, "immunogenicity" can be replaced with "antigenicity", because antigenicity refers to the antibody binding to the antigen, which was the focus of this study.
3. Page 3, line 65 – I recommend replacing "mPEG-liposomal mRNA" with "mPEG-containing lipid nanoparticles-mRNA". COVID vaccines do not use liposomes, they utilize lipid nanoparticles.
4. Page 3, line 65 – "can reduce unwanted uptake by the reticuloendothelial system in vivo" is an accurate statement about Doxil. However, it does not apply to LNP-mRNA COVID vaccines. In the lipid nanoparticles, the role of mPEG is to prevent LNP aggregation during storage as an aqueous dispersion.

5. Page 4, line 90 – I recommend replacing "generate" with either "induce" or "result in the generation of"

6. Page 5, line 98 – "PEG immunogenicity" should be "PEG antigenicity" because the study investigated structural aspects of anti-PEG antibody binding to its antigen (mPEG)

7. Page 16, line 289 – I recommend editing this sentence "mPEG is widely used in PEGylated medicine" for clarify. A possible alternative is to say "mPEG is widely used in medical products to improve solubility and circulation time of therapeutic proteins and nanoparticles"

8. Page 16, line 292 – "in many in naïve individuals" should be "in many naïve individuals"

Reviewer #3 (Remarks to the Author):

The manuscript describes the structural analysis of the first complex of the weak affinity, mouse-derived h15-2b antibody Fab fragment with m-PEG. The authors are to be commended for the crystal structure and the mutational analysis linked to activity, which define the critical residues and contact atoms used by the antibody. The work is of good quality and appears to have been diligently carried out.

It would have been good if the authors could have gone a step further to show how this structural information could be used to increase the affinity of a h15-2b-based PEG engager or assay reagent through rational design.

The abstract should be changed to highlight that the structure is of a complex, not just the Fab. This is very important, and currently missing.

Reviewer #4 (Remarks to the Author):

This manuscript entitled "Structural determination of an antibody that specifically recognizes polyethyleneglycol with a terminal methoxy group" by Nguyen et al. describes the crystal structure of h15-2b Fab in complex with mPEG along with supporting biochemical and mutagenesis data. The structural work was reasonably presented with some caveats described below. The biochemical data do support their notions in the discussion to certain extent although more data could be of great help.

Comments:
1. crystallography: The overall Rmerge of 21.3% and Rfree of 26% seem to be surprising high for data at this diffraction limits. This could indicate some pathologies in the data such as (pseudo)merohedral twinning, or that a lower-symmetry space group should have been used. The author should look into possible issues and review the crystallography work as needed, or at least provide sufficient justifications of these high values.
2. There are many close contacts of non-H atom pairs with clash distances between 0.4-0.7A (see validation report 5.2). The authors should carefully investigate these clashes and reduce this kind of clashes as much as possible.
3. P12-14, lines 211-242: The authors compared binding properties of mPEG, OH-PEG, and SH-PEH in the first Results section and Fig 1 (lines 102-124) and concluded the h15-2b Fab's mPEG specificity. Here, for the mutants data, the authors only compared variants binding between mPEG and OH-PEG. The data show some differences and the trends but not as convincing. To support the authors' arguments, it would be extremely helpful if similar data for SH-PEG can also be included in the manuscript. Is there a specific reason the authors did not present these data other than that the experiments were not conducted?

point-by-point response to the referees' comments

We appreciate the reviewer for taking the time to carefully review the manuscript and provided valuable comments to improve and clarify the manuscript. We have studied and address the comments point-by-point in below. Please find our responses and modifications written in blue and red, respectively.

Reviewer #1 (Remarks to the Author):

COMMSCHEM-22-0091

Title: Structural determination of an antibody that specifically recognizes polyethylene glycol with a terminal methoxy group.

In this research article, the authors build on previous work of elucidating the crystal structures of PEG-specific antibodies. In this article, they focus on antibody interactions with methoxy-PEG, using Fab molecules that are specific for the terminal group. The topic should be of interest to the community working on anti-PEG antibodies, and I found the mutagenesis data particularly interesting. I believe the work will be valuable for researchers trying to understand the nature of the interactions between antibodies and PEG. I suggest publication after minor revisions.

Specific comments:

1. I am not an expert in protein crystallization. However, I noticed that the authors crystallized the proteins in 18% OH-PEG-OH solutions (in the presence of 1% mPEG). I believe the authors should comment on the possibility of non-specific interactions between the protein and the OH-PEG-OH from the crystallization solvent. In other words, what precautions were taken to ensure that the structure computed from the X-ray crystallography experiment truly represents specific interactions with mPEG, and not serendipitous interactions with the (more abundant) PEG from the crystallization media? In previous published work, when the epitope was the backbone, this consideration might have been of lesser importance (the crystallization media would have the same backbone) – but to understand interactions with mPEG specifically, I believe clarifications might be in order.

We appreciate the reviewer to point out this issue. Although we determined the binding specificity of h15-2b towards mPEG but not OH-PEG in Figure 1, the more abundant OH-PEG might be a potential competitor. To further address this issue, we performed competitive ELISA to mimic the crystallization condition that covers the same molar ratio of OH-PEG_{6k} and $mPEG_{2k}$ (6:1) as shown in the modified

Supplementary Fig. S1. The competitive ELISA shows that h15-2b Fab can specifically bind to mPEG and tolerate the presence of 6-fold molar excess of OH-PEG_{6K}. Therefore, h15-2b is more likely to form a complex with $mPEG_{2K}$ instead of OH-PEG_{6K}.

We revised the Results section (h15-2b Fab Complex with mPEG deep in the V_H:V_L interface) to include this result on page 8 line 144.

"It should be noted that the co-crystal structure of h15-2b Fab and mPEG was crystallized by using a 6:1 molar ratio of OH-PEG_{6K} (15 mmol L^{-1}) and $mPEG_{2K}$ (2.5 mmol L^{-1}) as a cryoprotectant and an antigen, respectively. Although we demonstrated the specificity of h15-2b toward mPEG but not OH-PEG in Figure 1, it raises a question of whether h15-2b Fab forms a complex with $mPEG_{2K}$ or more abundant OH-PEG_{6K} during protein crystallization. To address this issue, we performed competitive ELISA with OH-PEG_{6K} and $mPEG_{2K}$. Microplates coated with $mPEG_{2K}$-NH_2 or TNF-α (2 μmol L^{-1}) were competed with either OH-PEG_{6K} or $mPEG_{5K}$ (12 μmol L^{-1}) in the presence of a fixed amount of Fab. We found that both OH-PEG_{6K} and $mPEG_{5K}$ blocked binding of 6.3 Fab (backbone-specific) to immobilized $mPEG_{2K}$-NH_2 in a dose-dependent manner (Supplementary Fig. S1a, b). By contrast, OH-PEG_{6K} is a poor competitor while $mPEG_{5K}$ is a strong competitor for binding of the h15-2b Fab (methoxy-specific) to immobilized $mPEG_{2K}$-NH_2. Therefore, the competitive ELISA results suggest that h15-2b Fab can specifically bind to mPEG and tolerate the presence of 6-fold molar excess of OH-PEG_{6K} (Supplementary Fig. S1). Furthermore, neither OH-PEG_{6K} nor $mPEG_{5K}$ affect the binding of an anti-TNF-α control Fab (Humira) to immobilized TNF-α, demonstrating the specificity of the competition reaction (Supplementary Fig. S1c, d). Taken together, h15-2b is more likely to form a complex with $mPEG_{2K}$ instead of OH-PEG_{6K}."

We re-organized Supplementary Fig. S1 in Supplementary Information. “Original Supplementary Fig. S1” was changed to “Supplementary Fig. S2”.

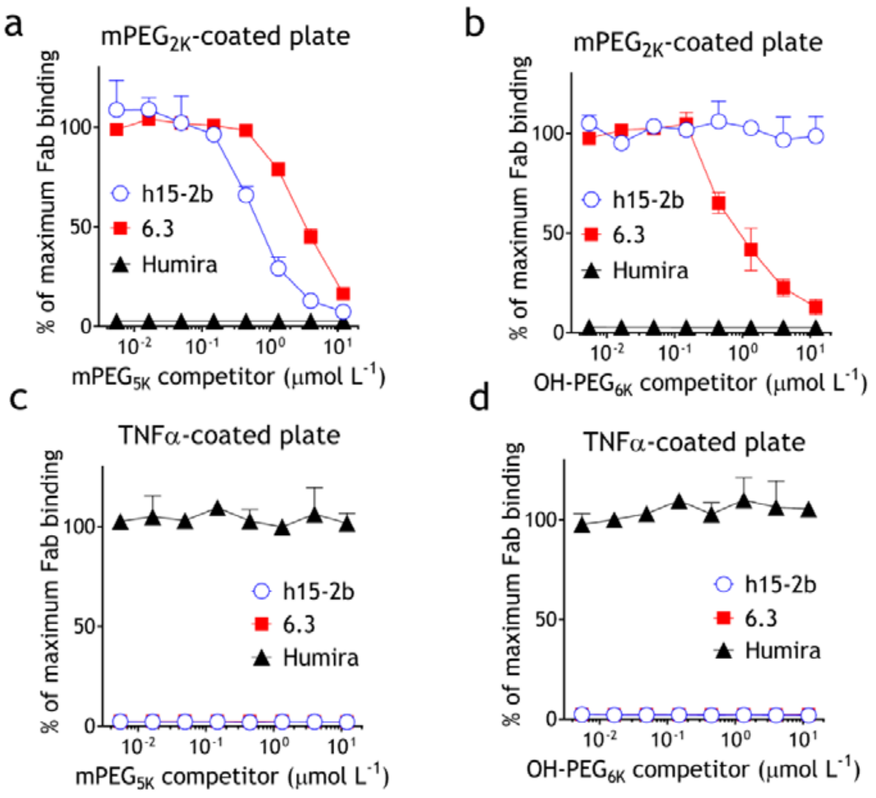

Supplementary Figure 1. The influence of excess OH-PEG in the anti-mPEG ELISA. Graded concentrations of competitors (OH-PEG_{6K} or $mPEG_{5K}$) were mixed with 0.5 μmol L^{-1} of h15-2b (open circle), 6.3 (closed square) or Humira (closed triangle) Fabs prior to addition to microplates coated with (**a, b**) $mPEG_{2K}$-NH_2 or (**c, d**) TNF-α. After washing, the Fab binding was determined by adding HRP-conjugated goat anti-human kappa light chain antibodies, followed by ABTS substrate. Results of triplicate determinations show Fab binding as a percentage of maximum binding activity. Bars, SD.

We revised the description in Supplementary Methods (Competitive ELISA). To use a uniform name of the secondary HRP-conjugated antibody, the “HRP-conjugated goat antihuman C kappa chain” was also changed to “HRP-conjugated goat anti-human kappa light chain” throughout this modified manuscript.

“Maxisorp 96-well microplates (Thermo Fisher Scientific, San Jose, CA) were coated with 2 μmol L^{-1} of $mPEG_{2k}$-NH_2 (Nanocs, New York, NY) or recombinant TNF-α (PeproTech, Cranbury, NJ) in 50 μL 100 mmol L^{-1} $NaHCO_3$/Na_2CO_3 coating buffer (pH 8.0) for 3 h at 37 °C and then blocked with 200 μL of 5% (wt/vol) skim milk in

PBS at 4 °C overnight. A fixed concentration of Fab (0.5 μmol L^{-1}) was mixed at a 1:1 volume ratio with graded concentrations (0.01–24 μmol L^{-1}) of OH-PEG_{6K} or $mPEG_{5K}$ (Sigma-Aldrich, St. Louis, MO) in 50 μL 2% (wt/vol) skim milk and then added to the plates for 1 h at room temperature. The plates were washed with PBS three times. HRP-conjugated goat anti-human kappa light chain antibodies (1 μg mL^{-1}) (Jackson Immuno Research Laboratories, West Grove, PA) in 50 μL 2% (wt/vol) skim milk were added for 30 min at room temperature. The plates were washed with PBS three times, and bound peroxidase activity was measured by adding 150 μL per well of ABTS substrate solution (0.4 mg mL^{-1} 2,2'-azino-di (3-ethylbenzthiazoline-6-sulfonic acid) (Sigma-Aldrich, St. Louis, MO), 0.003% H_2O_2, 100 mmol L^{-1} phosphate citrate, pH 4.0) for 30 min at room temperature. The absorbance (405 nm) was measured in a microplate reader (Molecular Device, Menlo Park, CA). Humira anti-TNF-α Fab was purchased from Rockland Immunochemicals (Pottstown, PA)."

2. I suggest the authors find a way of making the names of Fab variants more uniform. In some places (including figures), names are provided by using both the WT and mutant amino acids (Y91LW). Elsewhere, the mutants are referred to as "mutation of R100H to alanine". I believe reference between text and figures might be facilitated if the labelling was more uniform.

We revised the labeling of Fab variants to be uniform as shown below.

Page 11 line 198: "… the phenolic hydroxyl group of $Y36_L$ forms an H-bond…"

Page 12 line 212: "Unsurprisingly, mutation of $R101_H$ to alanine ($R101_HA$) completely abolished …"

Page 14 line 244: "… tyrosine or serine or threonine ($W104_HY$, $V37_HS$ and $V37_HT$) enhanced mPEG-binding and mildly increased binding …"

3. L87- The authors write: "PEG used in all therapeutic medicines is terminated by a methoxy group" – this statement should be revised: pegcetacoplan (Apellis pharma) is a pegylated peptide without a methoxy group, many drugs contain surfactants (notably polysorbate) that are not methoxy-terminated.

We revised the statement on page 4 line 87.

"Although PEG used in most therapeutic medicines is terminated by a methoxy group …"

4. I suggest the authors find a way of identifying the methoxy group in FIG2. Currently, the representation of the mPEG molecule has a carbon at both ends, which makes it unclear to fully understand where the end-group is.

We modified the labeling of methoxy group in the modified Fig. 2 on page10 line 164.

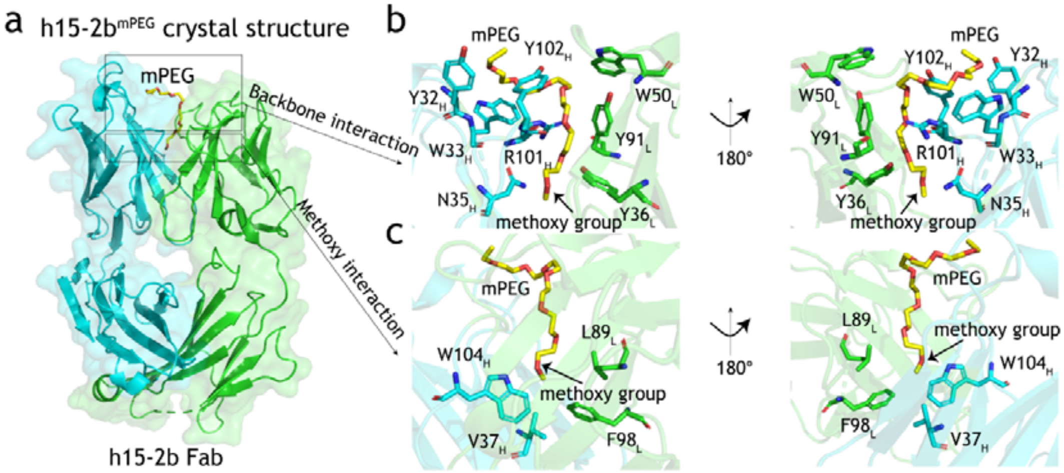

Fig. 2. Structural analysis of critical residues for the interaction of h15-2b Fab and mPEG. **a** Overall crystal structure of the mPEG molecule and anti-mPEG h15-2b Fab complex (Light chain in green; Heavy chain in cyan). The mPEG fragment bound by h15-2b Fab is shown as a yellow stick model. Superposition of (**b**) mPEG backbone-specific binding residues and (**c**) methoxy-specific binding residues in the h15-2b mPEG crystal structure are colored (Light chain in green; Heavy chain in cyan) and displayed in a transparent mode. The methoxy group of mPEG is indicated with an arrow.

5. L226 – In the sentence starting with "these results suggest…", it is unclear to me what 'these results' refer to.

We apologize for the unclear description of this sentence which was resulted in combination with a typing error. We revised it on page 14 line 244-247.

"Interestingly, mutating the hydrophobic W104$_H$ and V37$_H$ to a polar amino acid, tyrosine or serine or threonine (W104$_H$Y, V37$_H$S and V37$_H$T) enhanced mPEG-binding and mildly increased binding to OH-PEG, possibly by formation of hydrogen bonds to methoxy and hydroxyl groups on the mPEG and OH-PEG molecules, respectively (Fig.

4b). Taken together, Figure 4 reveals that hydrophilic amino acids (L89LD, F98LE, and W104HE) might reduce binding to mPEG and OH-PEG."

6. In the discussion, the authors summarize the hydrophobic residues that are important for mPEG binding (L267). It would be very interesting if the authors could compare what amino acids are present at these positions, on other antibodies that are backbone specific (i.e., not mPEG-specific).

Thank you for this important suggestion. In fact, these residues, including $V37_H$, $L89_L$, $F98_L$, and $W104_H$ are highly conserved throughout antibodies. After amino acid sequence alignment comparison (Supplementary Fig. S4), we found the main difference is that h15-2b possesses shorter heavy chain complementarity-determining region 3 (HCDR3) but similar light chain CDR3 (LCDR3) as compared to 3.3, 2B5 and 6.3.

We added the Supplementary Fig. S4 in the Supplementary Information.

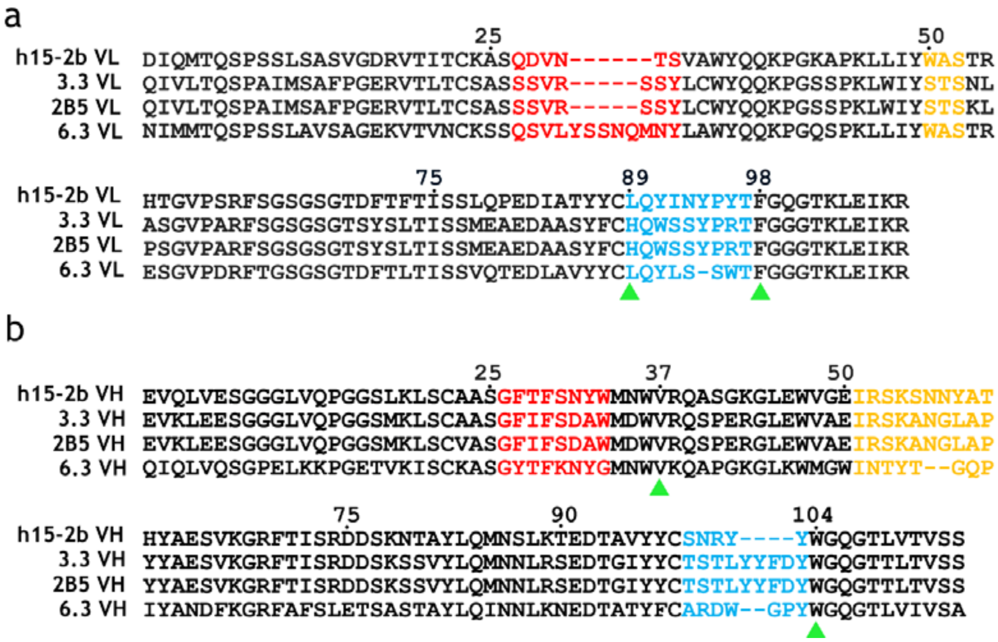

Supplementary Figure 4. Variable domain amino acid sequence alignments of anti-PEG and anti-mPEG antibodies. Amino acid sequence alignments of (**a**) variable light chain domain (V_L) and (**b**) Variable heavy chain domain (V_H) for anti-mPEG (h15-2b) and anti-PEG (3.3, 2B5 and 6.3) antibodies. The CDR regions are highlighted (CDR1, red; CDR2, yellow; CDR3, blue) and the conserved residues, including $L89_L$/$H89_L$, $F98_L$, $V37_H$ and $W104_H$ are indicated with green triangles.

We also added the description in Supplementary Methods (Amino acid sequence alignment of variable domains).

“The amino acid sequence alignment of variable domains of anti-PEG and anti-mPEG antibodies were compared by using Clustal Omega. Residues are numbered according to IMGT numbering and complementarity-determining regions CDRs were identified using the AbRSA online tool

.

We also performed a structural comparison focusing on the HCDR3 and LCDR3 of methoxy-specific and backbone-specific anti-PEG Fabs as shown in the modified Supplementary Fig. S5. We found that the HCDR3 loop of h15-2b helps to form a tunnel-like structure in the V_H:V_L interface. By contrast, the bulky HCDR3 loops of the backbone-specific anti-PEG Fabs hinder the formation of a tunnel-like structure and provide a large surface area to interact with the backbone of PEG chain. Overall, this might explain the unique mPEG-binding specificity of h15-2b.

We revised the description in the Discussion section on page 16 line 292.

“Note that although $V37_H$, $L89_L$, $F98_L$, and $W104_H$ residues are responsible for the methoxy-specificity of h15-2b, these residues are highly conserved throughout anti-PEG and anti-mPEG antibodies (Supplementary Fig. S4). We found that h15-2b possesses shorter heavy chain complementarity-determining region 3 (HCDR3) but similar light chain CDR3 (LCDR3) as compared to 3.3, 2B5 and 6.3 (Supplementary Fig. S4). Structural comparison of the anti-PEG and anti-mPEG Fabs reveals that the h15-2b HCDR3 loop coupled with these conserved residues assist to form a tunnel-like structure for capturing the mPEG deep in the V_H:V_L interface (Supplementary Fig. S5a). By contrast, the bulky HCDR3 loops of these anti-PEG Fabs hinder the tunnel-like structure formation and provide large surface area to interact with the backbone of PEG (Supplementary Fig. S5b-d). Therefore, this might explain the unique methoxy-specificity of h15-2b. “

We added the Supplementary Fig. S5 in the Supplementary Information.

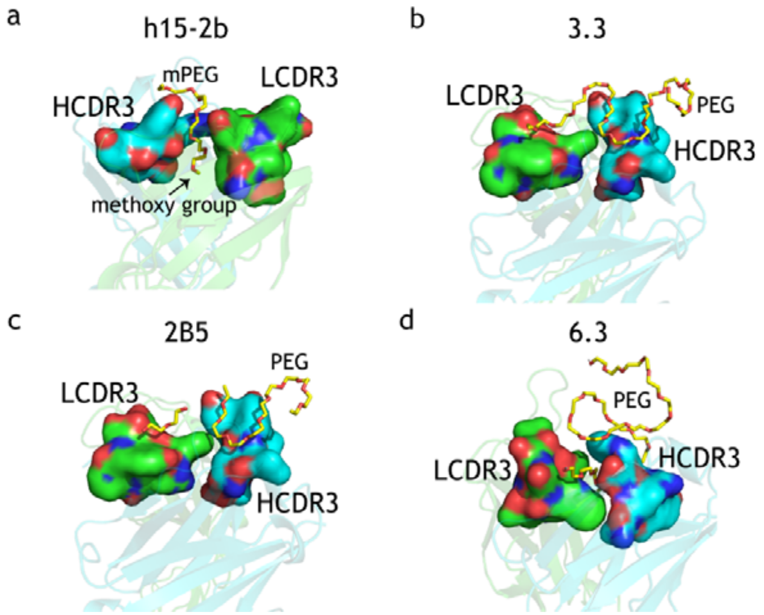

Supplementary Figure 5. Structural comparison of anti-mPEG and anti-PEG antibodies. **a** h15-2b; **b** 3.3; **c** 2B5; **d** 6.3. Surface representation of HCDR3 and LCDR3 are colored in cyan and green, respectively. Oxygen and nitrogen are colored in red and blue, respectively in the surface representation of CDRs. mPEG or PEG are colored yellow. The methoxy group of a mPEG is indicated with an arrow.

7. L271 – the authors write 'V37H plays an important role in 15-2b binding to methoxy terminated PEG'. From my understanding of the results, it seems like this amino acid is mostly important for selectivity toward methoxy-PEG (since mutants also seem to have higher affinity toward mPEG, In FIG4). I suggest correcting the sentence.

We correct the sentence on page 16 line 290-291

"… indicating that $V37_H$ is mostly important for selectivity of h15-2b toward methoxy terminated PEG."

Minor comments;

8. Legend of FIG 1C is SH-PEG3.5k-NH2 – the method section suggests PEG3.4k was used.

We used SH-$PEG_{3.5k}$-NH_2 from Sigma-Aldrich. We correct it in Methods section (Antibody ELISA) on page 23 line 428-430.

"… with 0.5 µg per well of $mPEG_{5k}$-NH_2 or OH-PEG_{5k}-NH_2 (Nanocs, New York, NY) or SH-$PEG_{3.5k}$-NH_2 (Sigma-Aldrich, St. Louis, MO) or recombinant GFP (Cell Biolabs, Inc., San Diego, CA) …"

9. L343 – The abbreviation PEG-2000-MME does not appear to be explained anywhere.

We correct it on page 21 line 389.

"… 1% (w/v) PEG-2000 methyl ether ..."

10. L354 – PDB abbreviation should be explained.

We correct it on page 21 line 400.

"… Fab2C4 (Protein Data Bank entry 1L7I) as a research model."

Reviewer #2 (Remarks to the Author):

The manuscript by Nguyen M.T. et al describes structural aspects of the interaction between anti-mPEG antibody and its putative antigen - mPEG. This study is of high value for the field of nanomedicine because mPEG is widely used in the preparation of nanocarriers used for drug delivery. The study is also of value for the therapeutic proteins field because mPEG is widely used to improve protein therapeutics' solubility and circulation time. One of the original goals of using mPEG in both nanomedicine and therapeutic proteins was to create a hydrophilic shield around nanoparticles or proteins to shield them from the immune system and prevent clearance by the MPS, also to decrease the immunogenicity of recombinant proteins. However, multiple studies have demonstrated that PEG and mPEG are immunogenic and their use in protein therapeutics and nanoparticles is associated with the formation of anti-PEG antibodies. Moreover, exposure to PEG early in life result is anti-PEG antibodies in the blood of healthy individuals. The antibodies against PEG contribute to hypersensitivity reactions and affect both pharmacokinetics and clearance of PEGylated products. However, a fundamental understanding of antigenicity - the binding of anti-PEG antibodies and their antigen (PEG) - is missing in the literature. This study fills this gap. I recommend it for publication after revision to address several points which I summarize below:

1. For the ELISA assay, the authors use Maxisorp plates and bi-functional PEG containing NH2-terminal groups on one end and mPEG terminal groups on another end. This approach may not provide optimal conditions for the orientation of mPEG on the plate, with some PEG molecules having a theoretical chance to be flat or bind to the plate exposing NH2-terminus. Did the authors try using plates with a specialized surface for binding amines? For example, https://cdn.gbiosciences.com/pdfs/protocol/Well-Coated_Amine_Binding_Plates.pdf If there was no difference between these plates and maxisorp plate, it may be stated in the materials section for clarification.

Thank you for this suggestion. We compared Maxisorp and amine-binding microplates. We found that there is no significant difference between these plates.

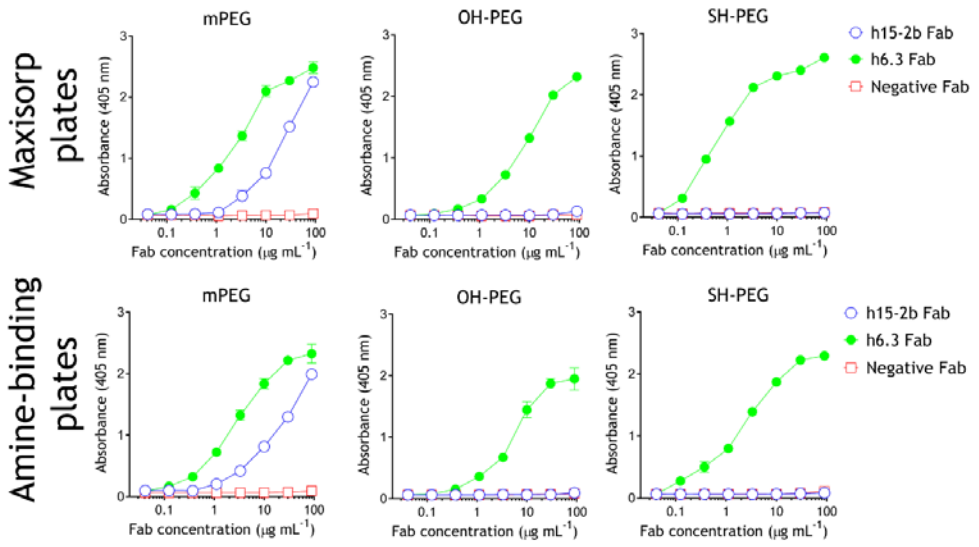

We added the description in the Methods section (Antibody ELISA) on page 24 line 443-448.

"Note that both Maxisorp (Thermo Fisher Scientific, San Jose, CA) and Well-Coated™ Amine Binding microplates (G-Biosciences, St. Louis, MO) are widely used for immobilization of amine group containing antigens. Comparison of these microplates for anti-PEG ELISA revealed that there is no significant difference between them. Therefore, the Maxisorp microplates were used for all ELISA experiments in this study."

2. Page 2, line 49 – I recommend removing "and immunogenicity of anti-PEG antibodies" because the study does not investigate PEG immunogenicity. The study

details structural aspects of the interaction between the antibody (anti-PEG antibody) and antigen (PEG molecule). Alternatively, "immunogenicity" can be replaced with "antigenicity", because antigenicity refers to the antibody binding to the antigen, which was the focus of this study.

We revised it on page 2 line 49.

"… understanding mPEG-binding specificity and antigenicity of anti-mPEG …"

3. Page 3, line 65 – I recommend replacing "mPEG-liposomal mRNA" with "mPEG-containing lipid nanoparticles-mRNA". COVID vaccines do not use liposomes, they utilize lipid nanoparticles.

Page 3, line 65 – "can reduce unwanted uptake by the reticuloendothelial system in vivo" is an accurate statement about Doxil. However, it does not apply to LNP-mRNA COVID vaccines. In the lipid nanoparticles, the role of mPEG is to prevent LNP aggregation during storage as an aqueous dispersion.

We modified it on page 3 line 64-67.

"… such as Doxil (mPEG-liposomal doxorubicin) and COVID-19 mRNA vaccines (mPEG-containing lipid nanoparticles-mRNA) can reduce unwanted uptake by the reticuloendothelial system *in vivo*[10] and prevent lipid nanoparticles-mRNA aggregation during storage as an aqueous dispersion[12], respectively."

4. Page 4, line 90 – I recommend replacing "generate" with either "induce" or "result in the generation of"

We revised it on page 4 line 89.

"… induce higher titers of anti-PEG antibodies …"

5. Page 5, line 98 – "PEG immunogenicity" should be "PEG antigenicity" because the study investigated structural aspects of anti-PEG antibody binding to its antigen (mPEG)

We revised it on page 5 line 98.

"…understanding of anti-PEG antibody specificity and mPEG antigenicity.…"

6. Page 16, line 289 – I recommend editing this sentence "mPEG is widely used in PEGylated medicine" for clarify. A possible alternative is to say "mPEG is widely used in medical products to improve solubility and circulation time of therapeutic proteins and nanoparticles"

We revised it on page 17 line 320-321.

"…mPEG is widely used in medical products to improve solubility and circulation time of therapeutic proteins and nanoparticles. Therefore, both …."

7. Page 16, line 292 – "in many in naïve individuals" should be "in many naïve individuals"

We revised it on page 18 line 323-324.

"… in many naïve individuals, the incidence of …"

Reviewer #3 (Remarks to the Author):

The manuscript describes the structural analysis of the first complex of the weak affinity, mouse-derived h15-2b antibody Fab fragment with m-PEG. The authors are to be commended for the crystal structure and the mutational analysis linked to activity, which define the critical residues and contact atoms used by the antibody. The work is of good quality and appears to have been diligently carried out.

1. It would have been good if the authors could have gone a step further to show how this structural information could be used to increase the affinity of a h15-2b-based PEG engager or assay reagent through rational design.

We appreciate the reviewer's suggestion. In this study, we mainly focused on structural analysis of the interaction between h15-2b and mPEG. Indeed, we found that some of the h15-2b variants such as $\mathrm{W104_{H}Y}$, $\mathrm{V37_{H}S}$ and $\mathrm{V37_{H}T}$ exhibit enhanced mPEG-binding as compared to wild-type h15-2b. Therefore, we also bring out some future research themes related to the improvement of the affinity of a h15-2b-based PEG engager or assay reagent in the "Discussion" section. In the future, we would like to investigate whether a combination of these mutations can create high-affinity h15-2b variants and extend the utility of these high-affinity h15-2b variants to develop the efficient targeted mPEG-nanomedicine.

To take this suggestion further, we have revised the Discussion on page 19 line 348-351.

"… to assist in the rational design of high-affinity anti-mPEG antibodies. This might be accomplished by the combination of the $W104_HY$, $V37_HS$ and $V37_HT$ mutations to create high-affinity h15-2b variants for the analytical study of pre-existing anti-mPEG antibodies and the development of efficient targeted mPEG-nanomedicine."

2. The abstract should be changed to highlight that the structure is of a complex, not just the Fab. This is very important, and currently missing.

Thank you for this suggestion. We revised the abstract on page 2 line 40-41.

"… Here, we determined the first co-crystal structure of the humanized 15-2b anti-mPEG antibody in complex with mPEG, which possesses a …"

Reviewer #4 (Remarks to the Author):

This manuscript entitled "Structural determination of an antibody that specifically recognizes polyethyleneglycol with a terminal methoxy group" by Nguyen et al. describes the crystal structure of h15-2b Fab in complex with mPEG along with supporting biochemical and mutagenesis data. The structural work was reasonably presented with some caveats described below. The biochemical data do support their notions in the discussion to certain extent although more data could be of great help.

Comments:
1. crystallography: The overall Rmerge of 21.3% and Rfree of 26% seem to be surprising high for data at this diffraction limits. This could indicate some pathologies in the data such as (pseudo)merohedral twinning, or that a lower-symmetry space group should have been used. The author should look into possible issues and review the crystallography work as needed, or at least provide sufficient justifications of these high values.

We have re-processed the diffraction data and re-deposited the structure on Protein Data Bank. The resolution of the new structure is improved to 2.08 Å. The R_{merge} and R_{free} are refined to 5.8% and 24.5%, respectively.

We revised the description in the Results section on page 7 line 130-134.

"… space group *P2₁* with the unit cell dimensions: a = 63.99 Å, b = 112.29 Å, c = 158.73 Å. The crystal structure of h15-2b^{mPEG} was determined by the molecular replacement (MR) method using the structure of the anti-ErbB2 Fab2C4 (Protein Data bank entry 1L7I) as the search model[28]. The final model of h15-2b^{mPEG} was refined to a resolution of 2.08 Å with an *R* factor of 21.1% and an R_{free} of 24.5%"

We also revised the description in Methods section to include this new Protein Data Bank ID on page 21 line 403 and page 24 line 453.

"… Fab/mPEG were deposited in the Protein Data Bank as entry 7Y0G"

We revised Table 1 as shown below.

Table 1. Crystallographic data and refinement statistics

**Structure**	**15-2b^{mPEG}**
**PDB ID**	7Y0G
**Data collection**	
Space group	$P2_1$
Cell dimensions	
a, *b*, *c* (Å)	63.99, 112.29, 158.73
α, β, γ (°)	90.00, 90.00, 90.00
Wavelength (Å)	0.9732
Resolution^a (Å)	30.00-2.08 (2.15-2.08)
R_{sym}^{b} or R_{merge}^{c} (%)	5.8 (49.2)
$CC_{1/2}$ (%)	96.7 (86.4)
I/σ	24.8 (2.7)
Completeness (%)	98.5 (93.1)
Redundancy	4.8 (4.4)
**Refinement**	
Resolution (Å)	30.00-2.08
No. reflections	131932
R_{work}/R_{free}	0.211/0.245
Ramachandran plot (%)^d	
favored	96.87
Outliers	0.24
***B*-factors**	
Protein	30.8
Ligand (mPEG)	38.4
Water	31.1
R.m.s deviations	
Bond lengths (Å)	0.0048
Bond angles (º)	1.358
Clashscore^e	2.59

^{a}Numbers in parentheses are values for the highest-resolution bin.

$^b R_{merge} = \sum_{hkl}\sum_i |I_i(hkl) - \bar{I}(hkl)| / \sum_{hkl}\sum_i I_i(hkl)$, where $I_i(hkl)$ is the *i*th observation of reflection *hkl* and $\bar{I}(hkl)$ is the weighted average intensity for all observations *i* of reflection *hkl*.

$^c R_{meas} = \sum_{hkl}[N/(N-1)^{1/2}]\sum_i |I_i(hkl) - \bar{I}(hkl)| / \sum_{hkl}\sum_i I_i(hkl)$.

^{d,e}As defined by MolProbity.

2. There are many close contacts of non-H atom pairs with clash distances between 0.4-0.7A (see validation report 5.2). The authors should carefully investigate these clashes and reduce this kind of clashes as much as possible.

We have re-refined the structure and lowered down the clashscore from 5 to 2. Although the clash distances of some atom pairs are still below 0.7 Å, the electron density map is clear to define these residues or water molecules.

3. P12-14, lines 211-242: The authors compared binding properties of mPEG, OH-PEG, and SH-PEH in the first Results section and Fig 1 (lines 102-124) and concluded the h15-2b Fab's mPEG specificity. Here, for the mutants data, the authors only compared variants binding between mPEG and OH-PEG. The data show some differences and the trends but not as convincing. To support the authors' arguments, it would be extremely helpful if similar data for SH-PEG can also be included in the manuscript. Is there a specific reason the authors did not present these data other than that the experiments were not conducted?

Thank you for pointing out this missing SH-PEG group. Indeed, we have the SH-PEG ELISA results and we should include this control group in our study. We found that all h15-2b variants are unable to bind to SH-PEG.

We modified the description in Results section (Characterization of important residues of h15-2b for mPEG-binding) on page 14 line 238-240.

"… from mPEG to OH-PEG or SH-PEG. None of the h15-2b variants bound to SH-PEG while L89LD, F98LE, and W104HE variants exhibited impaired binding to mPEG and OH-PEG (Supplementary Fig. S3; Fig. 4a; Supplementary Table S1)."

We modified the Supplementary Fig. S3 and Supplementary Table S1 to include the SH-PEG ELISA results.

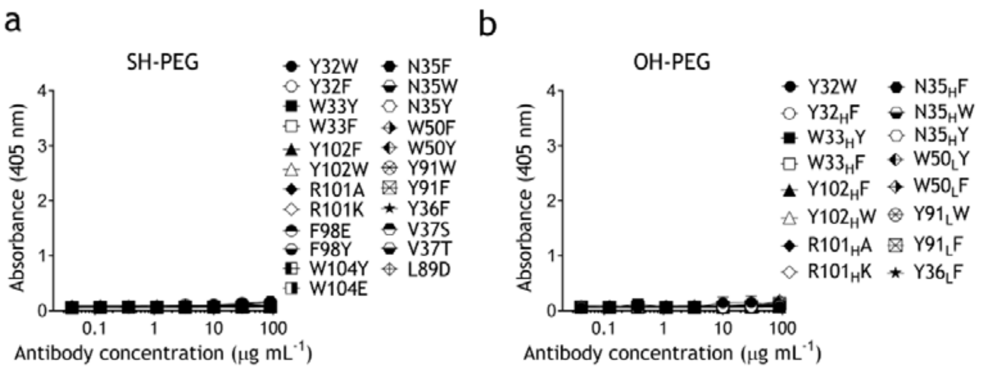

Supplementary Figure 3. Specificity of h15-2b Fab variants against thiol-PEG and hydroxyl-PEG molecules. Microplate wells coated with (**a**) $SH\text{-}PEG_{3.5K}\text{-}NH_2$ or (**b**) $OH\text{-}PEG_{5K}\text{-}NH_2$ were incubated with graded concentrations of h15-2b variants. After 1h, the wells were washed, and antibody binding was determined by adding HRP-conjugated goat anti-human kappa light chain antibodies, followed by ABTS substrate. The mean absorbance values (405 nm) of triplicate determinations are shown. Bars, SD.

Supplementary Table 1. The relative sensitives of anti-mPEG antibody variants

The relative sensitivities of h15-2b anti-mPEG antibody variants in ELISA

Variants	mPEG-binding sensitivity[a]	OH-PEG-binding sensitivity[a]	SH-PEG-binding sensitivity[a]
WT	2.3	NB	NB
$Y32_HF$	4.3	NB	NB
$Y32_HW$	34.6	NB	NB
$Y102_HF$	1.6	NB	NB
$Y102_HW$	7.6	NB	NB
$W33_HF$	28.6	NB	NB
$W33_HY$	6.9	NB	NB
$W50_LF$	4.8	NB	NB
$W50_LY$	1.8	NB	NB
$N35_HF$	NB	NB	NB
$N35_HY$	NB	NB	NB
$N35_HW$	NB	NB	NB
$Y36_LF$	92.6	NB	NB
$Y91_LF$	3.1	NB	NB
$Y91_LW$	3.1	NB	NB
$R101_HA$	NB	NB	NB
$R101_HK$	NB	NB	NB
$L89_LD$	NB	NB	NB
$F98_LE$	30.7	NB	NB
$F98_LY$	4.5	NB	NB
$W104_HY$	0.79	42.7	NB
$W104_HE$	43.2	NB	NB
$V37_HS$	0.6	20.5	NB
$V37_HT$	1.1	14.9	NB

[a] Results show the concentration of antibody (μg mL^{-1}) that can produce an absorbance reading (A405) of 0.5 in an ELISA using the indicated antibody variants. [b] NB, no binding, baseline reading at all concentrations.

Reviewers' comments:

Reviewer #1 (Remarks to the Author):

The authors have addressed all of the reviewers' comments. The resulting article is very interesting, and I believe the manuscript is now suitable for publication.

Reviewer #2 (Remarks to the Author):

The authors have addressed my comments, and I recommend the revised manuscript for publication. This study is urgently needed as many scientists in the nanotechnology community doubt the existence of the anti-PEG antibodies because they do not understand how such antibodies bind to PEG, and literature on the topic is rather scarse.

Reviewer #4 (Remarks to the Author):

Regarding authors' responses to Comment 1. Crystallography:

The authors did discover that their data should be processed in a space group with lower symmetry, In this new space group, given similar cell constants, there should be twice amount of molecules in the asymmetric unit than that in their original cell. However, throughout the manuscript, the authors did not spell out how many copies of Fab molecules and how many mPEGs are in the asymmetric unit (i.e. in the PDB file they deposited). The authors also should address how similar and how dissimilar these copies of Fabs are in terms of root-mean-square deviations and to what extent these differences affect their findings and conclusions. These are basic contents in a "Structural determination" paper to provide a solid base to show that the structural features are indeed well beyond coordinate deviations (or not). Also, for figures showing structures (Fig 2,3,4), the authors should clearly state which chains are shown in each figure and other copies of chains of similar nature (e.g. VL, VH, or mPEG…) in the PDB file behave similarly or not.

point-by-point response to the referees' comments

We appreciate the reviewer for taking the time to carefully review the manuscript and provided valuable comments to improve and clarify the manuscript. We have studied and address the comments point-by-point in below. Please find our responses and modifications written in blue and red, respectively.

Reviewer #1 (Remarks to the Author):

COMMSCHEM-22-0091

Title: Structural determination of an antibody that specifically recognizes polyethylene glycol with a terminal methoxy group.

In this research article, the authors build on previous work of elucidating the crystal structures of PEG-specific antibodies. In this article, they focus on antibody interactions with methoxy-PEG, using Fab molecules that are specific for the terminal group. The topic should be of interest to the community working on anti-PEG antibodies, and I found the mutagenesis data particularly interesting. I believe the work will be valuable for researchers trying to understand the nature of the interactions between antibodies and PEG. I suggest publication after minor revisions.

Specific comments:

1. I am not an expert in protein crystallization. However, I noticed that the authors crystallized the proteins in 18% OH-PEG-OH solutions (in the presence of 1% mPEG). I believe the authors should comment on the possibility of non-specific interactions between the protein and the OH-PEG-OH from the crystallization solvent. In other words, what precautions were taken to ensure that the structure computed from the X-ray crystallography experiment truly represents specific interactions with mPEG, and not serendipitous interactions with the (more abundant) PEG from the crystallization media? In previous published work, when the epitope was the backbone, this consideration might have been of lesser importance (the crystallization media would have the same backbone) – but to understand interactions with mPEG specifically, I believe clarifications might be in order.

We appreciate the reviewer to point out this issue. Although we determined the binding specificity of h15-2b towards mPEG but not OH-PEG in Figure 1, the more abundant OH-PEG might be a potential competitor. To further address this issue, we performed competitive ELISA to mimic the crystallization condition that covers the same molar ratio of OH-PEG_{6k} and $mPEG_{2k}$ (6:1) as shown in the modified

Supplementary Fig. S2. The competitive ELISA shows that h15-2b Fab can specifically bind to mPEG and tolerate the presence of 6-fold molar excess of OH-PEG_{6K}. Therefore, h15-2b is more likely to form a complex with $mPEG_{2K}$ instead of OH-PEG_{6K}.

We revised the Results section (h15-2b Fab Complex with mPEG deep in the V_H:V_L interface) to include this result on page 8 line 156.

"It should be noted that the co-crystal structure of h15-2b Fab and mPEG was crystallized by using a 6:1 molar ratio of OH-PEG_{6K} (15 mmol L^{-1}) and $mPEG_{2K}$ (2.5 mmol L^{-1}) as a cryoprotectant and an antigen, respectively. Although we demonstrated the specificity of h15-2b toward mPEG but not OH-PEG in Figure 1, it raises a question of whether h15-2b Fab forms a complex with $mPEG_{2K}$ or more abundant OH-PEG_{6K} during protein crystallization. To address this issue, we performed competitive ELISA with OH-PEG_{6K} and $mPEG_{2K}$. Microplates coated with $mPEG_{2K}$-NH_2 or TNF-α (2 μmol L^{-1}) were competed with either OH-PEG_{6K} or $mPEG_{5K}$ (12 μmol L^{-1}) in the presence of a fixed amount of Fab. We found that both OH-PEG_{6K} and $mPEG_{5K}$ blocked binding of 6.3 Fab (backbone-specific) to immobilized $mPEG_{2K}$-NH_2 in a dose-dependent manner (Supplementary Fig. S2a, b). By contrast, OH-PEG_{6K} is a poor competitor while $mPEG_{5K}$ is a strong competitor for binding of the h15-2b Fab (methoxy-specific) to immobilized $mPEG_{2K}$-NH_2. Therefore, the competitive ELISA results suggest that h15-2b Fab can specifically bind to mPEG and tolerate the presence of 6-fold molar excess of OH-PEG_{6K} (Supplementary Fig. S2). Furthermore, neither OH-PEG_{6K} nor $mPEG_{5K}$ affect the binding of an anti-TNF-α control Fab (Humira) to immobilized TNF-α, demonstrating the specificity of the competition reaction (Supplementary Fig. S2c, d). Taken together, h15-2b is more likely to form a complex with $mPEG_{2K}$ instead of OH-PEG_{6K}."

We re-organized Supplementary Fig. S2 in Supplementary Information. "Original Supplementary Fig. S1" was changed to "Supplementary Fig. S3".

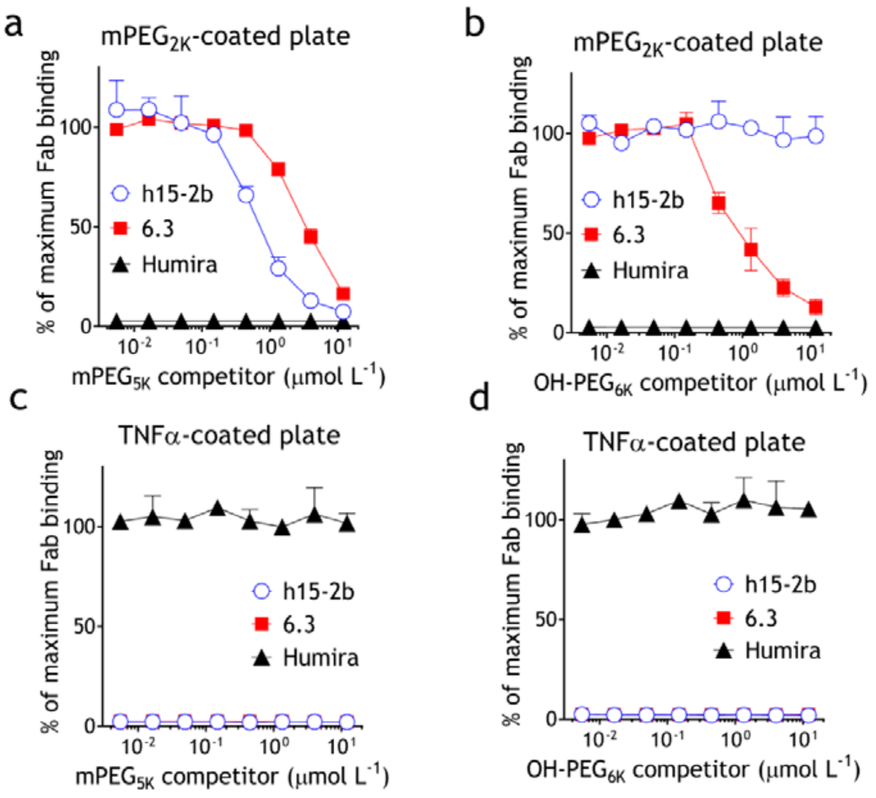

Supplementary Figure 2. The influence of excess OH-PEG in the anti-mPEG ELISA. Graded concentrations of competitors (OH-PEG_{6K} or $mPEG_{5K}$) were mixed with 0.5 μmol L^{-1} of h15-2b (open circle), 6.3 (closed square) or Humira (closed triangle) Fabs prior to addition to microplates coated with (**a, b**) $mPEG_{2K}$-NH_2 or (**c, d**) TNF-α. After washing, the Fab binding was determined by adding HRP-conjugated goat anti-human kappa light chain antibodies, followed by ABTS substrate. Results of triplicate determinations show Fab binding as a percentage of maximum binding activity. Bars, SD. The data are representative of three independent experiments.

We revised the description in Supplementary Methods (Competitive ELISA). To use a uniform name of the secondary HRP-conjugated antibody, the "HRP-conjugated goat antihuman C kappa chain" was also changed to "HRP-conjugated goat anti-human kappa light chain" throughout this modified manuscript.

"Maxisorp 96-well microplates (Thermo Fisher Scientific, San Jose, CA) were coated with 2 μmol L^{-1} of $mPEG_{2k}$-NH_2 (Nanocs, New York, NY) or recombinant TNF-α (PeproTech, Cranbury, NJ) in 50 μL 100 mmol L^{-1} $NaHCO_3$/Na_2CO_3 coating buffer (pH 8.0) for 3 h at 37 °C and then blocked with 200 μL of 5% (wt/vol) skim milk in

PBS at 4 °C overnight. A fixed concentration of Fab (0.5 μmol L^{-1}) was mixed at a 1:1 volume ratio with graded concentrations (0.01–24 μmol L^{-1}) of OH-PEG_{6K} or $mPEG_{5K}$ (Sigma-Aldrich, St. Louis, MO) in 50 μL 2% (wt/vol) skim milk and then added to the plates for 1 h at room temperature. The plates were washed with PBS three times. HRP-conjugated goat anti-human kappa light chain antibodies (1 μg mL^{-1}) (Jackson Immuno Research Laboratories, West Grove, PA) in 50 μL 2% (wt/vol) skim milk were added for 30 min at room temperature. The plates were washed with PBS three times, and bound peroxidase activity was measured by adding 150 μL per well of ABTS substrate solution (0.4 mg mL^{-1} 2,2'-azino-di (3-ethylbenzthiazoline-6-sulfonic acid) (Sigma-Aldrich, St. Louis, MO), 0.003% H_2O_2, 100 mmol L^{-1} phosphate citrate, pH 4.0) for 30 min at room temperature. The absorbance (405 nm) was measured in a microplate reader (Molecular Device, Menlo Park, CA). Humira anti-TNF-α Fab was purchased from Rockland Immunochemicals (Pottstown, PA)."

2. I suggest the authors find a way of making the names of Fab variants more uniform. In some places (including figures), names are provided by using both the WT and mutant amino acids (Y91LW). Elsewhere, the mutants are referred to as "mutation of R100H to alanine". I believe reference between text and figures might be facilitated if the labelling was more uniform.

We revised the labeling of Fab variants to be uniform as shown below.

Page 12 line 210: "… the phenolic hydroxyl group of $Y36_L$ forms an H-bond…"

Page 13 line 224: "Unsurprisingly, mutation of $R101_H$ to alanine ($R101_HA$) completely abolished …"

Page 15 line 256: "… tyrosine or serine or threonine ($W104_HY$, $V37_HS$ and $V37_HT$) enhanced mPEG-binding and mildly increased binding …"

3. L87- The authors write: "PEG used in all therapeutic medicines is terminated by a methoxy group" – this statement should be revised: pegcetacoplan (Apellis pharma) is a pegylated peptide without a methoxy group, many drugs contain surfactants (notably polysorbate) that are not methoxy-terminated.

We revised the statement on page 4 line 87.

"Although PEG used in most therapeutic medicines is terminated by a methoxy group …"

4. I suggest the authors find a way of identifying the methoxy group in FIG2. Currently, the representation of the mPEG molecule has a carbon at both ends, which makes it unclear to fully understand where the end-group is.

We modified the labeling of methoxy group in the modified Fig. 2 on page10 line 175.

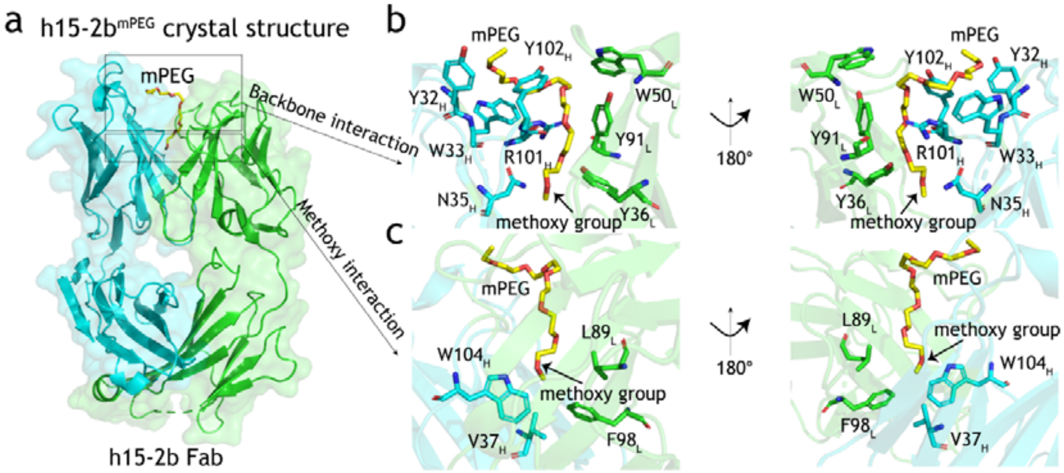

Fig. 2. Structural analysis of critical residues for the interaction of h15-2b Fab and mPEG. **a** Overall crystal structure of the mPEG molecule and anti-mPEG h15-2b Fab complex (Light chain in green; Heavy chain in cyan). The mPEG fragment bound by h15-2b Fab is shown as a yellow stick model. Superposition of (**b**) mPEG backbone-specific binding residues and (**c**) methoxy-specific binding residues in the h15-2b mPEG crystal structure are colored (Light chain in green; Heavy chain in cyan) and displayed in a transparent mode. The methoxy group of mPEG is indicated with an arrow.

5. L226 – In the sentence starting with "these results suggest…", it is unclear to me what 'these results' refer to.

We apologize for the unclear description of this sentence which was resulted in combination with a typing error. We revised it on page 15 line 256-259.

"Interestingly, mutating the hydrophobic W104$_{H}$ and V37$_{H}$ to a polar amino acid, tyrosine or serine or threonine (W104$_{H}$Y, V37$_{H}$S and V37$_{H}$T) enhanced mPEG-binding and mildly increased binding to OH-PEG, possibly by formation of hydrogen bonds to methoxy and hydroxyl groups on the mPEG and OH-PEG molecules, respectively (Fig.

4b). Taken together, Figure 4 reveals that hydrophilic amino acids (L89LD, F98LE, and W104HE) might reduce binding to mPEG and OH-PEG."

6. In the discussion, the authors summarize the hydrophobic residues that are important for mPEG binding (L267). It would be very interesting if the authors could compare what amino acids are present at these positions, on other antibodies that are backbone specific (i.e., not mPEG-specific).

Thank you for this important suggestion. In fact, these residues, including V37H, L89L, F98L, and W104H are highly conserved throughout antibodies. After amino acid sequence alignment comparison (Supplementary Fig. S5), we found the main difference is that h15-2b possesses shorter heavy chain complementarity-determining region 3 (HCDR3) but similar light chain CDR3 (LCDR3) as compared to 3.3, 2B5 and 6.3.

We added the Supplementary Fig. S5 in the Supplementary Information.

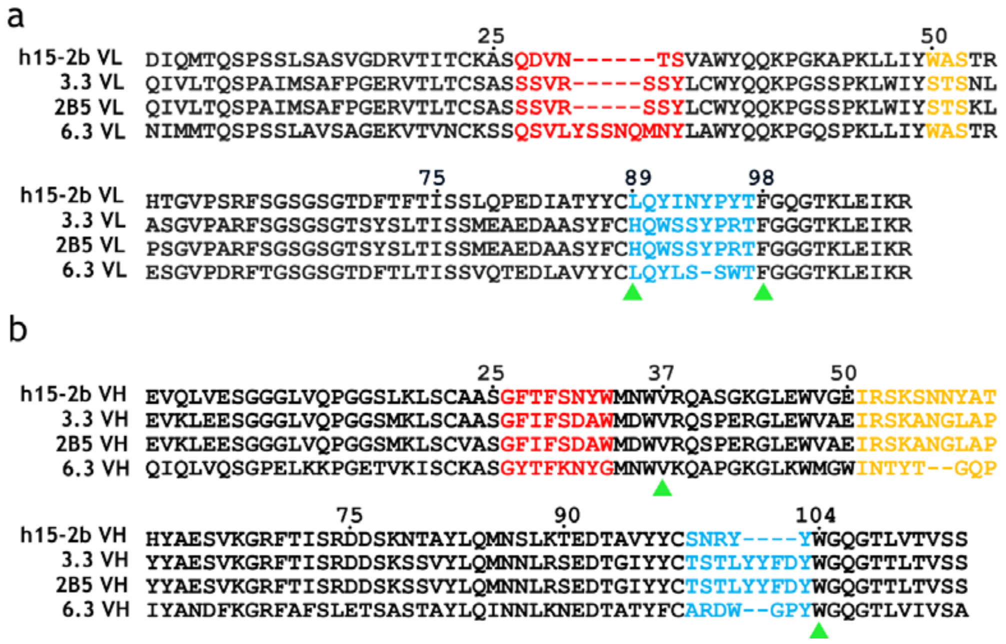

Supplementary Figure 5. Variable domain amino acid sequence alignments of anti-PEG and anti-mPEG antibodies. Amino acid sequence alignments of (**a**) variable light chain domain (VL) and (**b**) Variable heavy chain domain (VH) for anti-mPEG (h15-2b) and anti-PEG (3.3, 2B5 and 6.3) antibodies. The CDR regions are highlighted (CDR1, red; CDR2, yellow; CDR3, blue) and the conserved residues, including L89L/H89L, F98L, V37H and W104H are indicated with green triangles.

We also added the description in Supplementary Methods (Amino acid sequence alignment of variable domains).

“The amino acid sequence alignment of variable domains of anti-PEG and anti-mPEG antibodies were compared by using Clustal Omega. Residues are numbered according to IMGT numbering and complementarity-determining regions CDRs were identified using the AbRSA online tool

.

We also performed a structural comparison focusing on the HCDR3 and LCDR3 of methoxy-specific and backbone-specific anti-PEG Fabs as shown in the modified Supplementary Fig. S6. We found that the HCDR3 loop of h15-2b helps to form a tunnel-like structure in the V_H:V_L interface. By contrast, the bulky HCDR3 loops of the backbone-specific anti-PEG Fabs hinder the formation of a tunnel-like structure and provide a large surface area to interact with the backbone of PEG chain. Overall, this might explain the unique mPEG-binding specificity of h15-2b.

We revised the description in the Discussion section on page 17 line 298.

“Note that although $V37_H$, $L89_L$, $F98_L$, and $W104_H$ residues are responsible for the methoxy-specificity of h15-2b, these residues are highly conserved throughout anti-PEG and anti-mPEG antibodies (Supplementary Fig. S5). We found that h15-2b possesses shorter heavy chain complementarity-determining region 3 (HCDR3) but similar light chain CDR3 (LCDR3) as compared to 3.3, 2B5 and 6.3 (Supplementary Fig. S5). Structural comparison of the anti-PEG and anti-mPEG Fabs reveals that the h15-2b HCDR3 loop coupled with these conserved residues assist to form a tunnel-like structure for capturing the mPEG deep in the V_H:V_L interface (Supplementary Fig. S6a). By contrast, the bulky HCDR3 loops of these anti-PEG Fabs hinder the tunnel-like structure formation and provide large surface area to interact with the backbone of PEG (Supplementary Fig. S6b-d). Therefore, this might explain the unique methoxy-specificity of h15-2b. “

We added the Supplementary Fig. S6 in the Supplementary Information.

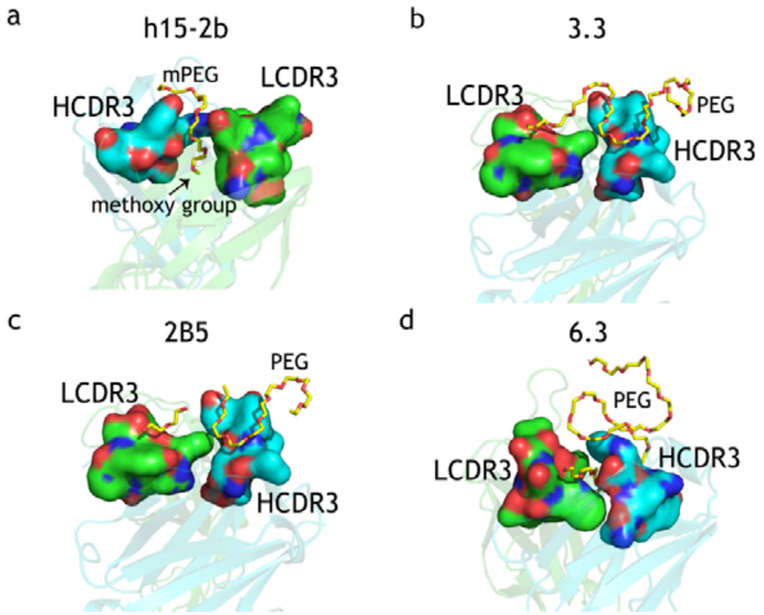

Supplementary Figure 6. Structural comparison of anti-mPEG and anti-PEG antibodies. **a** h15-2b; **b** 3.3; **c** 2B5; **d** 6.3. Surface representation of HCDR3 and LCDR3 are colored in cyan and green, respectively. Oxygen and nitrogen are colored in red and blue, respectively in the surface representation of CDRs. mPEG or PEG are colored yellow. The methoxy group of a mPEG is indicated with an arrow.

7. L271 – the authors write 'V37H plays an important role in 15-2b binding to methoxy terminated PEG'. From my understanding of the results, it seems like this amino acid is mostly important for selectivity toward methoxy-PEG (since mutants also seem to have higher affinity toward mPEG, In FIG4). I suggest correcting the sentence.

We correct the sentence on page 17 line 296-297

"… indicating that $V37_H$ is mostly important for selectivity of h15-2b toward methoxy terminated PEG."

Minor comments;
8. Legend of FIG 1C is SH-PEG3.5k-NH2 – the method section suggests PEG3.4k was used.

We used $SH\text{-}PEG_{3.5K}\text{-}NH_2$ from Sigma-Aldrich. We correct it in Methods section (Antibody ELISA) on page 24 line 434-436.

"… with 0.5 µg per well of $mPEG_{5k}$-NH_2 or OH-PEG_{5K}-NH_2 (Nanocs, New York, NY) or SH-$PEG_{3.5K}$-NH_2 (Sigma-Aldrich, St. Louis, MO) or recombinant GFP (Cell Biolabs, Inc., San Diego, CA) …"

9. L343 – The abbreviation PEG-2000-MME does not appear to be explained anywhere.

We correct it on page 22 line 395.

"… 1% (w/v) PEG-2000 methyl ether ..."

10. L354 – PDB abbreviation should be explained.

We correct it on page 22 line 406.

"… Fab2C4 (Protein Data Bank entry 1L7I) as a research model."

Reviewer #2 (Remarks to the Author):

The manuscript by Nguyen M.T. et al describes structural aspects of the interaction between anti-mPEG antibody and its putative antigen - mPEG. This study is of high value for the field of nanomedicine because mPEG is widely used in the preparation of nanocarriers used for drug delivery. The study is also of value for the therapeutic proteins field because mPEG is widely used to improve protein therapeutics' solubility and circulation time. One of the original goals of using mPEG in both nanomedicine and therapeutic proteins was to create a hydrophilic shield around nanoparticles or proteins to shield them from the immune system and prevent clearance by the MPS, also to decrease the immunogenicity of recombinant proteins. However, multiple studies have demonstrated that PEG and mPEG are immunogenic and their use in protein therapeutics and nanoparticles is associated with the formation of anti-PEG antibodies. Moreover, exposure to PEG early in life result is anti-PEG antibodies in the blood of healthy individuals. The antibodies against PEG contribute to hypersensitivity reactions and affect both pharmacokinetics and clearance of PEGylated products. However, a fundamental understanding of antigenicity - the binding of anti-PEG antibodies and their antigen (PEG) - is missing in the literature. This study fills this gap. I recommend it for publication after revision to address several points which I summarize below:

1. For the ELISA assay, the authors use Maxisorp plates and bi-functional PEG containing NH2-terminal groups on one end and mPEG terminal groups on another end. This approach may not provide optimal conditions for the orientation of mPEG on the plate, with some PEG molecules having a theoretical chance to be flat or bind to the plate exposing NH2-terminus. Did the authors try using plates with a specialized surface for binding amines? For example, https://cdn.gbiosciences.com/pdfs/protocol/Well-Coated_Amine_Binding_Plates.pdf If there was no difference between these plates and maxisorp plate, it may be stated in the materials section for clarification.

Thank you for this suggestion. We compared Maxisorp and amine-binding microplates. We found that there is no significant difference between these plates.

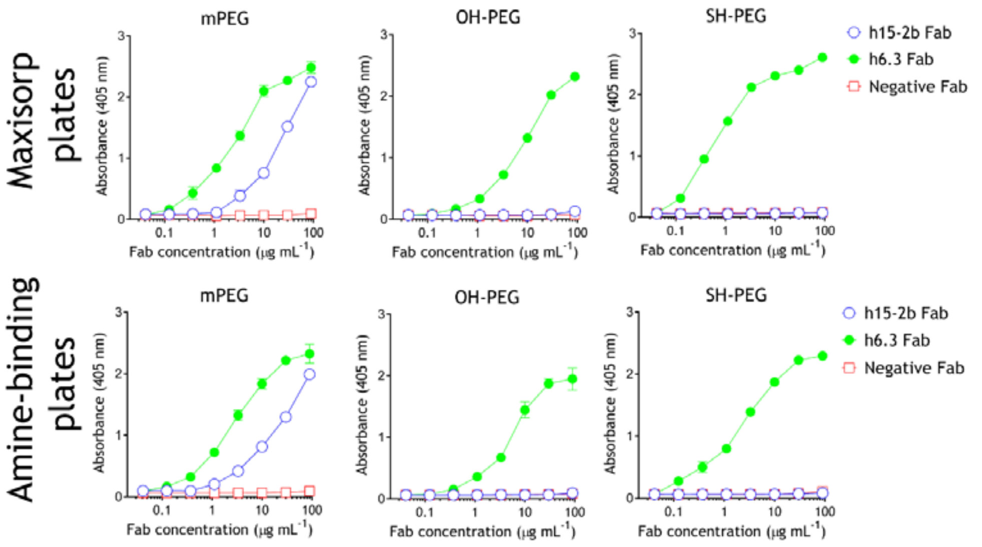

We added the description in the Methods section (Antibody ELISA) on page 25 line 449-454.

"Note that both Maxisorp (Thermo Fisher Scientific, San Jose, CA) and Well-Coated™ Amine Binding microplates (G-Biosciences, St. Louis, MO) are widely used for immobilization of amine group containing antigens. Comparison of these microplates for anti-PEG ELISA revealed that there is no significant difference between them. Therefore, the Maxisorp microplates were used for all ELISA experiments in this study."

2. Page 2, line 49 – I recommend removing "and immunogenicity of anti-PEG antibodies" because the study does not investigate PEG immunogenicity. The study

details structural aspects of the interaction between the antibody (anti-PEG antibody) and antigen (PEG molecule). Alternatively, "immunogenicity" can be replaced with "antigenicity", because antigenicity refers to the antibody binding to the antigen, which was the focus of this study.

We revised it on page 2 line 49.

"… understanding mPEG-binding specificity and antigenicity of anti-mPEG …"

3. Page 3, line 65 – I recommend replacing "mPEG-liposomal mRNA" with "mPEG-containing lipid nanoparticles-mRNA". COVID vaccines do not use liposomes, they utilize lipid nanoparticles.

Page 3, line 65 – "can reduce unwanted uptake by the reticuloendothelial system in vivo" is an accurate statement about Doxil. However, it does not apply to LNP-mRNA COVID vaccines. In the lipid nanoparticles, the role of mPEG is to prevent LNP aggregation during storage as an aqueous dispersion.

We modified it on page 3 line 64-67.

"… such as Doxil (mPEG-liposomal doxorubicin) and COVID-19 mRNA vaccines (mPEG-containing lipid nanoparticles-mRNA) can reduce unwanted uptake by the reticuloendothelial system *in vivo*[10] and prevent lipid nanoparticles-mRNA aggregation during storage as an aqueous dispersion[12], respectively."

4. Page 4, line 90 – I recommend replacing "generate" with either "induce" or "result in the generation of"

We revised it on page 4 line 89.

"… induce higher titers of anti-PEG antibodies …"

5. Page 5, line 98 – "PEG immunogenicity" should be "PEG antigenicity" because the study investigated structural aspects of anti-PEG antibody binding to its antigen (mPEG)

We revised it on page 5 line 98.

"…understanding of anti-PEG antibody specificity and mPEG antigenicity.…"

6. Page 16, line 289 – I recommend editing this sentence "mPEG is widely used in PEGylated medicine" for clarify. A possible alternative is to say "mPEG is widely used in medical products to improve solubility and circulation time of therapeutic proteins and nanoparticles"

We revised it on page 18 line 326-327.

"…mPEG is widely used in medical products to improve solubility and circulation time of therapeutic proteins and nanoparticles. Therefore, both .…"

7. Page 16, line 292 – "in many in naïve individuals" should be "in many naïve individuals"

We revised it on page 19 line 329.

"… in many naïve individuals, the incidence of …"

Reviewer #3 (Remarks to the Author):

The manuscript describes the structural analysis of the first complex of the weak affinity, mouse-derived h15-2b antibody Fab fragment with m-PEG. The authors are to be commended for the crystal structure and the mutational analysis linked to activity, which define the critical residues and contact atoms used by the antibody. The work is of good quality and appears to have been diligently carried out.

1. It would have been good if the authors could have gone a step further to show how this structural information could be used to increase the affinity of a h15-2b-based PEG engager or assay reagent through rational design.

We appreciate the reviewer's suggestion. In this study, we mainly focused on structural analysis of the interaction between h15-2b and mPEG. Indeed, we found that some of the h15-2b variants such as $W104_{H}Y$, $V37_{H}S$ and $V37_{H}T$ exhibit enhanced mPEG-binding as compared to wild-type h15-2b. Therefore, we also bring out some future research themes related to the improvement of the affinity of a h15-2b-based PEG engager or assay reagent in the "Discussion" section. In the future, we would like to investigate whether a combination of these mutations can create high-affinity h15-2b variants and extend the utility of these high-affinity h15-2b variants to develop the efficient targeted mPEG-nanomedicine.

To take this suggestion further, we have revised the Discussion on page 20 line 354-357.

"… to assist in the rational design of high-affinity anti-mPEG antibodies. This might be accomplished by the combination of the $W104_HY$, $V37_HS$ and $V37_HT$ mutations to create high-affinity h15-2b variants for the analytical study of pre-existing anti-mPEG antibodies and the development of efficient targeted mPEG-nanomedicine."

2. The abstract should be changed to highlight that the structure is of a complex, not just the Fab. This is very important, and currently missing.

Thank you for this suggestion. We revised the abstract on page 2 line 40-41.

"… Here, we determined the first co-crystal structure of the humanized 15-2b anti-mPEG antibody in complex with mPEG, which possesses a …"

Reviewer #4 (Remarks to the Author):

This manuscript entitled "Structural determination of an antibody that specifically recognizes polyethyleneglycol with a terminal methoxy group" by Nguyen et al. describes the crystal structure of h15-2b Fab in complex with mPEG along with supporting biochemical and mutagenesis data. The structural work was reasonably presented with some caveats described below. The biochemical data do support their notions in the discussion to certain extent although more data could be of great help.

Comments:
1. crystallography: The overall Rmerge of 21.3% and Rfree of 26% seem to be surprising high for data at this diffraction limits. This could indicate some pathologies in the data such as (pseudo)merohedral twinning, or that a lower-symmetry space group should have been used. The author should look into possible issues and review the crystallography work as needed, or at least provide sufficient justifications of these high values.

We have re-processed the diffraction data and re-deposited the structure on Protein Data Bank. The resolution of the new structure is improved to 2.08 Å. The R_{merge} and R_{free} are refined to 5.8% and 24.5%, respectively.

We revised the description in the Results section on page 7 line 131-135.

"… space group *P2₁* with the unit cell dimensions: a = 63.99 Å, b = 112.29 Å, c = 158.73 Å. The crystal structure of h15-2b^{mPEG} was determined by the molecular replacement (MR) method using the structure of the anti-ErbB2 Fab2C4 (Protein Data bank entry 1L7I) as the search model[28]. The final model of h15-2b^{mPEG} was refined to a resolution of 2.08 Å with an *R* factor of 21.1% and an R_{free} of 24.5%"

We also revised the description in Methods section to include this new Protein Data Bank ID on page 22 line 409 and page 25 line 459.

"… Fab/mPEG were deposited in the Protein Data Bank as entry 7Y0G"

We revised Table 1 as shown below.

Table 1. Crystallographic data and refinement statistics

**Structure**	**15-2b^{mPEG}**
**PDB ID**	7Y0G
**Data collection**	
Space group	$P2_1$
Cell dimensions	
a, *b*, *c* (Å)	63.99, 112.29, 158.73
α, β, γ (°)	90.00, 90.00, 90.00
Wavelength (Å)	0.9732
Resolution^a (Å)	30.00-2.08 (2.15-2.08)
$R_{sym}{}^b$ or $R_{merge}{}^c$ (%)	5.8 (49.2)
$CC_{1/2}$ (%)	96.7 (86.4)
I/σ	24.8 (2.7)
Completeness (%)	98.5 (93.1)
Redundancy	4.8 (4.4)
**Refinement**	
Resolution (Å)	30.00-2.08
No. reflections	131932
R_{work}/R_{free}	0.211/0.245
Ramachandran plot (%)^d	
favored	96.87
Outliers	0.24
***B*-factors**	
Protein	30.8
Ligand (mPEG)	38.4
Water	31.1
R.m.s deviations	
Bond lengths (Å)	0.0048
Bond angles (°)	1.358
Clashscore^e	2.59

[a]Numbers in parentheses are values for the highest-resolution bin.
[b]$R_{merge} = \sum_{hkl}\sum_i|I_i(hkl) - \bar{I}(hkl)|/\sum_{hkl}\sum_i I_i(hkl)$, where $I_i(hkl)$ is the *i*th observation of reflection *hkl* and $\bar{I}(hkl)$ is the weighted average intensity for all observations *i* of reflection *hkl*.
[c]$R_{meas} = \sum_{hkl}[N/(N-1)^{1/2}]\sum_i|I_i(hkl) - \bar{I}(hkl)|/\sum_{hkl}\sum_i I_i(hkl)$.
[d,e]As defined by MolProbity.

2. There are many close contacts of non-H atom pairs with clash distances between 0.4-0.7A (see validation report 5.2). The authors should carefully investigate these clashes and reduce this kind of clashes as much as possible.

We have re-refined the structure and lowered down the clashscore from 5 to 2. Although the clash distances of some atom pairs are still below 0.7 Å, the electron density map is clear to define these residues or water molecules.

3. P12-14, lines 211-242: The authors compared binding properties of mPEG, OH-PEG, and SH-PEH in the first Results section and Fig 1 (lines 102-124) and concluded the h15-2b Fab's mPEG specificity. Here, for the mutants data, the authors only compared variants binding between mPEG and OH-PEG. The data show some differences and the trends but not as convincing. To support the authors' arguments, it would be extremely helpful if similar data for SH-PEG can also be included in the manuscript. Is there a specific reason the authors did not present these data other than that the experiments were not conducted?

Thank you for pointing out this missing SH-PEG group. Indeed, we have the SH-PEG ELISA results and we should include this control group in our study. We found that all h15-2b variants are unable to bind to SH-PEG.

We modified the description in Results section (Characterization of important residues of h15-2b for mPEG-binding) on page 15 line 250-252.

"… from mPEG to OH-PEG or SH-PEG. None of the h15-2b variants bound to SH-PEG while L89LD, F98LE, and W104HE variants exhibited impaired binding to mPEG and OH-PEG (Supplementary Fig. S4; Fig. 4a; Supplementary Table S1)."

We modified the Supplementary Fig. S4 and Supplementary Table S1 to include the SH-PEG ELISA results.

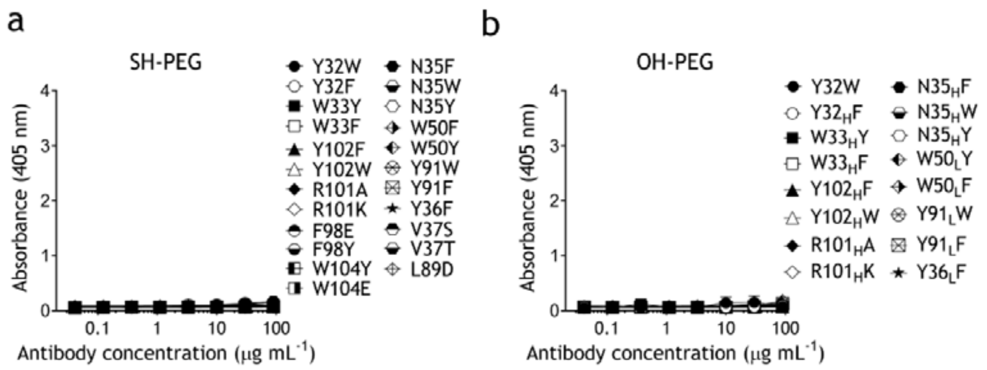

Supplementary Figure 4. Specificity of h15-2b Fab variants against thiol-PEG and hydroxyl-PEG molecules. Microplate wells coated with (**a**) SH-$PEG_{3.5K}$-NH_2 or (**b**) OH-PEG_{5K}-NH_2 were incubated with graded concentrations of h15-2b variants. After 1h, the wells were washed, and antibody binding was determined by adding HRP-conjugated goat anti-human kappa light chain antibodies, followed by ABTS substrate. The mean absorbance values (405 nm) of triplicate determinations are shown. Bars, SD. The data are representative of three independent experiments.

Supplementary Table 1. The relative sensitives of anti-mPEG antibody variants

The relative sensitivities of h15-2b anti-mPEG antibody variants in ELISA			
Variants	mPEG-binding sensitivity[a]	OH-PEG-binding sensitivity[a]	SH-PEG-binding sensitivity[a]
WT	2.3	NB	NB
$Y32_HF$	4.3	NB	NB
$Y32_HW$	34.6	NB	NB
$Y102_HF$	1.6	NB	NB
$Y102_HW$	7.6	NB	NB
$W33_HF$	28.6	NB	NB
$W33_HY$	6.9	NB	NB
$W50_LF$	4.8	NB	NB
$W50_LY$	1.8	NB	NB
$N35_HF$	NB	NB	NB
$N35_HY$	NB	NB	NB
$N35_HW$	NB	NB	NB
$Y36_LF$	92.6	NB	NB
$Y91_LF$	3.1	NB	NB
$Y91_LW$	3.1	NB	NB
$R101_HA$	NB	NB	NB
$R101_HK$	NB	NB	NB
$L89_LD$	NB	NB	NB
$F98_LE$	30.7	NB	NB
$F98_LY$	4.5	NB	NB
$W104_HY$	0.79	42.7	NB
$W104_HE$	43.2	NB	NB
$V37_HS$	0.6	20.5	NB
$V37_HT$	1.1	14.9	NB

[a] Results show the concentration of antibody (μg mL^{-1}) that can produce an absorbance reading (A405) of 0.5 in an ELISA using the indicated antibody variants. [b] NB, no binding, baseline reading at all concentrations.

4. Regarding authors' responses to Comment 1. Crystallography:

The authors did discover that their data should be processed in a space group with lower symmetry, In this new space group, given similar cell constants, there should be twice amount of molecules in the asymmetric unit than that in their original cell. However, throughout the manuscript, the authors did not spell out how many copies of Fab molecules and how many mPEGs are in the asymmetric unit (i.e. in the PDB file they deposited). The authors also should address how similar and how dissimilar these copies of Fabs are in terms of root-mean-square deviations and to what extent these differences affect their findings and conclusions. These are basic contents in a "Structural determination" paper to provide a solid base to show that the structural features are indeed well beyond coordinate deviations (or not). Also, for figures showing structures (Fig 2,3,4), the authors should clearly state which chains are shown in each figure and other copies of chains of similar nature (e.g. VL, VH, or mPEG…) in the PDB file behave similarly or not.

We thank the reviewer to point out this issue. The structure of h15-2b^{mPEG} contains four copies of h15-2b Fab in an asymmetric unit, which all share nearly identical structures with small root-mean-square deviations (rmsds) of 0.161–0.417 Å for Cα atom superposition (Supplementary Fig. S1a). The superposition of the four copies of h15-2b Fab reveals that the mPEG fragments binding poses are almost identical (Supplementary Fig. S1b). In this study, one of the h15-2b Fab structures (Chain A for light chain and Chain B for heavy chain in the atomic coordinates of h15-2b^{mPEG}) was utilized for figure generation (Fig. 2-4).

We added the description in the Results section.

page 7 line 136: "The structure of h15-2b^{mPEG} contains four copies of h15-2b Fab in an asymmetric unit, which all share nearly identical structures with small root-mean-square deviations (rmsds) of 0.161–0.417 Å for Ca atom superposition (Supplementary Fig. S1a). The light chains of the four copies of h15-2b Fab had varying electron density from the N- to C-termini, with the start from the first residue and ending between residues 211 and 213. The heavy chains of the four copies of h15-2b Fab could be reliably traced between residues 1 and 214–216, with the region between residues 127 and 135 lacking significant density. Each of h15-2b Fabs shows that…"

page8 line 146-147: "The superposition of the four copies of h15-2b Fab reveals that the binding poses of the mPEG fragments are almost identical (Supplementary Fig.

S1b).”

page 8 line 152-155:” In this study, one of the h15-2b Fab structures (Chain A for light chain and Chain B for heavy chain in the atomic coordinates of h15-2b^{mPEG}) was utilized for figure generation (Fig.2-4).”

We added the Supplementary Fig. S1 in the Supplementary Information

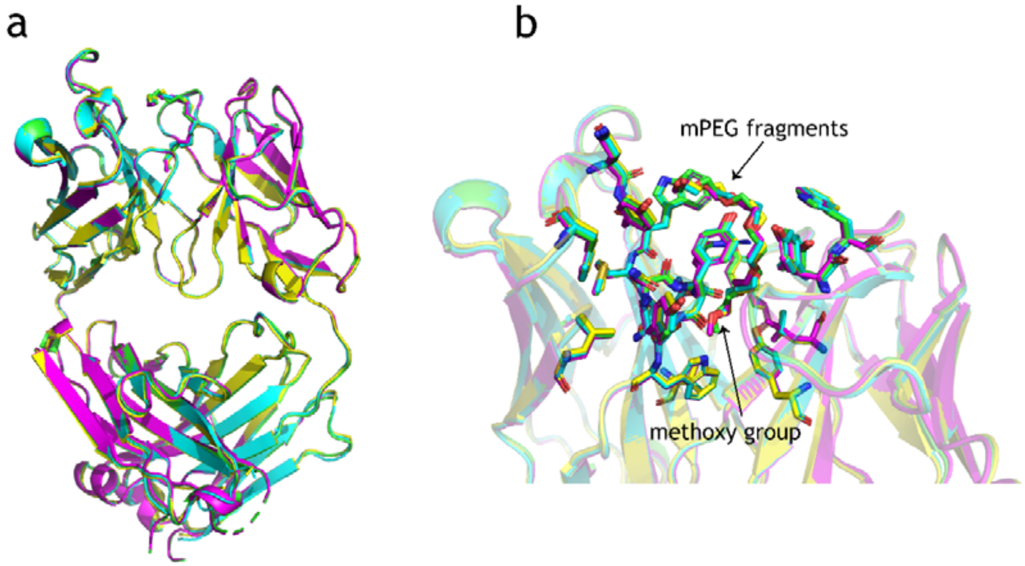

Supplementary Figure 1. The crystal structures of the four copies of h15-2b Fabs. **a** Superposition of the overall structures of the four copies of h15-2b Fabs. **b** Local view of each mPEG binding site in the four copies of h15-2b Fabs. The four copies of h15-2b and mPEG fragments are colored green, cyan, magentas, and yellow, respectively.

REVIEWERS' COMMENTS:

Reviewer #4 (Remarks to the Author):

The authors have addressed my comments.
